# Resolving a QTL complex for height, heading, and grain yield on chromosome 3A in bread wheat

**DOI:** 10.1093/jxb/erab058

**Published:** 2021-02-12

**Authors:** Alba Farre Martinez, Clare Lister, Sue Freeman, Jun Ma, Simon Berry, Luzie Wingen, Simon Griffiths

**Affiliations:** 1 John Innes Centre, Colney Lane, Norwich NR4 7UH, UK; 2 College of Agronomy and Biotechnology, China Agricultural University, Beijing, 100094, China; 3 Limagrain UK, Woolpit Business Park, Woolpit, Bury St Edmunds, Suffolk,UK; 4 CSIRO Agriculture and Food, Australia

**Keywords:** Avalon, Cadenza, cell wall, earliness *per se*, flowering, height, QTL, wheat, yield

## Abstract

Crop height (Ht), heading date (Hd), and grain yield (GY) are inter-related in wheat. Independent manipulation of each is important for adaptation and performance. Validated quantitative trait loci (QTLs) for all three co-locate on chromosome 3A in the Avalon×Cadenza population, with increased Ht, Hd, and GY contributed by Cadenza. We asked if these are linked or pleiotropic effects using recombinant lines, and showed that Ht and Hd effects are independent. The Chinese Spring equivalent to the newly defined Ht interval contained a gene cluster involved in cell wall growth and displaying high levels of differential transcript expression. The Hd locus is larger and rearranged compared with the reference genome, but *FT2* (*Flowering Locus T2*) is of particular interest. The Hd effect acted independently of photoperiod and vernalization, but did exhibit seasonal genotype×environment interaction. Recombinants were phenotyped for GY in replicated field experiments. GY was most associated with Cadenza alleles for later Hd, supporting physiological studies using the same lines proposing that ‘late’ alleles at this locus increase spike fertility and grain number (GN). The work has uncoupled height from heading and yield, and shown that one of very few validated GY QTLs in wheat is probably mediated by phenological variation.

## Teaser

There are only three validated wheat yield QTLs. Here, one of them was genetically dissected. This showed that the physiological basis of the yield effect is likely to be phenological.

## Introduction

Breeding for increased grain yield (GY) is achieved together with the optimization of other important agronomic traits, notably height (Ht) and heading date (Hd). While the direction of selection for GY is always for an increase, the other two traits are adaptive, which means that the optimum Ht and Hd of the crop depend very much on the target environment. This presents a challenge because all three traits are often related. For example, selection for yield alone can result in tall (Aisawi *et al.*, 2015) late heading lines (Bogard *et al.*, 2011) which are prone to lodging, late season stress, and delayed harvest. It is also the case that competitive traits such as crop height can result in phantom yield effects in small plot experiments largely as a result of edge effects (Rebetzke *et al.*, 2014) which are not translated into any on-farm yield benefit but, again, increase vulnerability to lodging. It is very important that these physiological and genetic relationships are understood.

A small number of major genes have been used by breeders to mould their germplasm into primary adaptive classes targeting broad environmental categories such as those captured in the mega-environment concept (Rajaram *et al.*, 1993). So, for Ht, major semi-dwarfing genes are deployed such as those for reduced height, *Rht-B1b*, *Rht-D1b* (Miralles and Slafer, 1995), and *Rht8* (Worland *et al.*, 1998*b*). For Hd, photoperiod and vernalization sensitivity genes are used in a similar way, with the consequence that allelic classes for groups of major genes are often fixed, or at least represented at a high frequency, within germplasm pools targeting specific mega-environments (Kiss *et al.*, 2014). For photoperiod response these are mainly homoeoalleles of *Ppd-1* (Worland *et al.*, 1998*a*) and *Ppd-2* (Zikhali *et al.*, 2017), and, for vernalization, mainly *Vrn-1* (Iwaki *et al.*, 2001),

After these major gene combinations have been optimized, genetic progress for adaptation, and its interplay with grain yield, made within these pools depends on polygenic variation comprising individual effects which are relatively small and identified as quantitative trait loci (QTLs). More detailed investigation into these effects, often relying on their conversion to discrete Mendelian factors by the production of near isogenic lines (NILs), facilitates a deeper understanding of the function(s) of gene(s) underlying the QTLs which are being used by breeders in that programme. In this way, NIL development facilitated the characterization of a heading date QTL identified on 1DL as an earliness *per se* (*eps*) effect (Zikhali *et al.*, 2014) which was then described as a discrete Mendelian factor called *EPS-D1* caused by deletion of *ELF3* (Zikhali *et al.*, 2016). Analysis of floral development showed that the late alleles of *EPS-D1* increased spike fertility through enhanced floret survival (Prieto *et al.*, 2018). Controlled environments were used to show that *EPS-D1* displayed a strong interaction with ambient temperature variation (Ochagavía *et al.*, 2019). A similar depth of understanding of all major QTLs segregating in elite germplasm pools is an important foundation stone for future genetic gains in genomics-led breeding programmes.

Through the use of segregating populations or association panels derived from varieties which are well adapted to the same environment, a number of studies have been able to identify the QTLs that are being used to achieve incremental yield gains and fine tuning of adaptation. The Avalon×Cadenza (A×C) doubled haploid (DH) population was used by us as part of a meta-QTL study to describe variation in UK wheat for Hd (Griffiths *et al.*, 2009), Ht (Griffiths *et al.*, 2012), and GY (Ma *et al.*, 2015). A library of NILs was then produced in which reciprocal transfers of Avalon and Cadenza alleles into the opposing variety allowed QTL validation in both parental contexts (Farré *et al.*, 2016). This allowed us to validate the original A×C QTLs and prioritize key effects. A QTL identified on chromosome 3A was of particular interest as the same locus affected GY, Ht, and Hd, with the Cadenza allele increasing all three. Very few robust GY QTLs have been identified in wheat and an even smaller number validated by comparison of NILs. These include a grain size effect on chromosome 6A that increases GY (Simmonds *et al.*, 2014) and *Rht-1* semi-dwarfing which increases yield via increased GN in some environments (Miralles and Slafer, 1995). The A×C 3A GY QTLs and derived NILs increase GY through GN (Farré *et al.*, 2016). This is important because almost all of the genetic gains achieved for GY and GY plasticity in wheat have been as a consequence of increased GN (Slafer *et al.*, 2014).

It was not known whether the Hd and Ht effects co-located with GY at the 3A locus are associated by genetic linkage or pleiotropy. The aim of this study is to show whether they are genetically distinct, increase understanding of the mechanism to show how they might contribute to adaptation and GY, and develop assays facilitating precise marker-assisted selection at this locus.

## Materials and methods

### Development of NILs and recombinants

The A×C DH population was one of several developed to represent a broad spectrum of the variation present in the UK elite winter germplasm pool and is now the UK reference population under the UK Department of Environment, Food and Rural Affairs (DEFRA) Wheat Genetic Improvement Network (WGIN). Several Hd, Ht, and GY QTLs have been previously identified in the A×C DH population (Griffiths *et al.*, 2009, 2012; Ma *et al.*, 2015). In this work we are focused on the Hd, Ht, and GY QTLs located on chromosome 3AS. Both Avalon (UK winter wheat) and Cadenza (UK alternative wheat) carry photoperiod-sensitive alleles of *Ppd-D1* and *Ppd-B1*. Avalon carries the winter alleles of *vrn-A1*, *vrn-B1*, and *vrn-D1*, whereas Cadenza carries a dominant *Vrn-A1a* allele conferring a facultative/spring growth habit. Avalon is a semi-dwarf carrying *Rht-D1b*, while Cadenza carries *Rht-D1a* (wild type).

The development of families of NILs and their use for the validation of the 3A Hd, Ht, and GY QTLs is described in Farré *et al.* (2016). In this work, a single Cadenza background NIL pair from that study is used for further analysis; they are designated NIL-A (carrying the Avalon 3A introgression) and NIL-C (carring the Cadenza 3A introgression). Thus, NIL-C is genetically very similar to Cadenza but with ~12% random Avalon background. For the development of recombinants within the introgressed segment, BC_2_ (BC_3_ equivalent because the backcross donor parent was a line from the A×C segregating DH population) heterozygous plants from the same backcross lineage and NIL-A and NIL-C were self-pollinated. Lines heterozygous for a region between simple sequence repeat (SSR) markers *wmc505* and *wmc264*, identified as a meta-QTL region for flowering on 3A in Griffiths *et al.* (2009), were self-pollinated to generate a BC_2_F_3_ of 454 individuals. These markers were then used to identify recombinants in this interval. Eighty-four of these BC_2_F_3_ recombinants were self-pollinated, and homozygous BC_2_F_4_ recombinants were selected using the two flanking SSR markers. A total of 76 recombinant BC_2_F_4_ lines were selected for further analysis.

### Assessing photoperiod sensitivity of 3A heading date effect

The A×C BC_2_ NILs were grown under controlled environments. Seeds were sown in January 2014 and grown in an unheated but daylength-controlled glasshouse and therefore fully vernalized at 6–10 °C using natural vernalization, under short days (SDs, 10 h light) for 8 weeks. The plants were then grown at 13–18 °C under two photoperiod treatments, SDs or long days (LDs, 16 h light). Plants under SDs and LDs were grown with natural light for 10 h and the LD plants had an additional artificially extended photoperiod of 6 h using tungsten bulbs. We used eight 60 W tungsten lamps spaced 90 cm apart and 2.1 m above the plants, delivering 1 µmol s^–1^ m^–2^. The plants were grown in a randomized complete block design with three replicates. NILs were classified according to their genotype across the *wmc505*–*wmc264* genetic interval (24 with the Avalon and 19 with the Cadenza introgression). To verify that the NILs had been adequately vernalized, five plants each of the winter wheat cultivars Claire, Malacca, and Hereward were grown as controls. Hereward flowers >30 d later than Malacca and Claire when incompletely vernalized for 4 weeks, and this was associated with copy number variation at *Vrn-A1* (Diaz *et al*., 2012). Days to ear emergence (Hd) was scored as the number of days after 28 April when the ear was >50% emerged from the flag leaf on the main shoot and corresponding to Zadoks stage 55 (Zadoks *et al.*, 1974). Data were evaluated using two-way ANOVA in which the interaction between treatment and allele was included in the model. ANOVA was performed using Genstat 16th edition (VSN International).

### Assessing vernalization sensitivity of 3A heading date effect

For the controlled-environment experiments, 32 out of 76 chromosome 3A recombinants were selected based on the extent of the Avalon introgression. The plants were grown under different vernalization treatments (0, 4, 6, and 8 weeks under SDs at 6 °C) and then transferred to the LD photoperiod (as described above). The plants were grown in a randomized complete block design with three replicates for each treatment. The mixed model used included treatment, allele, and the interaction between treatment and allele as fixed factors, and blocks as a random factor. Blocks were considered random factors in the model in order to recover interblock information due to the presence of missing values. The mixed model was fitted using linear mixed procedures from the Genstat 16th edition (VSN International). Heading date was recorded as days to ear emergence. In this case, Hd indicates the difference between days from 50% ear emergence and the day that plants were transferred from the vernalization treatment to LDs. The winter wheat cultivars Claire, Malacca, and Hereward were also grown under these conditions as controls.

### Measurement of developmental phases

The NILs (NIL-A and NIL-C) were used to determine which developmental phases were affected by the 3A QTL. NIL-A (carrying the Avalon introgression in the QTL region) and NIL-C (carrying the Cadenza introgression) came from the AC179-E27-2 stream (Farré *et al.*, 2016). Plants were vernalized for 8 weeks at 6 °C under SDs and then transferred to a glasshouse with a temperature around 18 °C and LD photoperiod. Apices were dissected from three randomly selected plants of each NIL every 2–3 d and examined under a light microscope. This allowed the time from sowing to double ridge (S–DR), double ridge to terminal spikelet (DR–TS), and from terminal spikelet to heading (TS–HD), to be determined, following the scales proposed by Kirby and Appleyard (1987). Hd was scored as above. Booting and tiller number were also recorded.

### Statistical analysis of GY

A simple linear model (function lm in R version 3.6.1) was fitted to analyse the relationship between a trait and the genetic markers in the QTL region. *P*-values were calculated from the *t*-statistics. Box-plots were plotted using the function boxplot in R.

### Phenotype evaluation

Seventy-six recombinant BC_2_F_4_ lines were phenotyped in two field experiments (spring sown and autumn sown) and under controlled environments. Field trials were conducted at Church Farm, Bawburgh, Norfolk, UK, (52°37′51.6′′N, 1°10′38.6′′E) in 2013 (spring and autumn sown). Details of meteorological conditions are given in Supplementary Fig. S1. Experimental design followed a randomized complete block design with three replicates. Plots consisted of four rows, 1 m long and 12 cm apart, and grown according to standard agricultural practice, except that plant growth regulators (PGRs) were not applied. The experiment included both parents (Avalon and Cadenza) and NIL-A and NIL-C in each block. Hd was assessed in thermal time (°C d, using a base temperature of 0 °C). Ht was measured from soil level to the tip of the ear (cm). None of the material was awned so this is not a complicating feature in the description of height.

### Genetic mapping and QTL analysis

Genomic DNA extraction was performed using published protocols adapted from Pallota *et al*. (2003). To increase marker resolution across the 3A QTL region, 65 additional markers were chosen. These were mainly KASP (kompetitive allele-specific PCR) markers selected from the integrated 3A genetic map at CerealsDB (http://www.cerealsdb.uk.net/cerealgenomics/CerealsDB/kasp_mapped_snps.php) or KASP markers derived from iSelect markers (Avni *et al.*, 2014). Marker information can be found at CerealsDB (http://www.cerealsdb.uk.net/cerealgenomics/CerealsDB/iselect_mapped_snps.php).

Methods for genotyping with the SSR and KASP assays used have been described previously in Wingen *et al.* (2014) and Zikhali *et al.* (2014), with the precise conditions used dependent on the specific primer pairs. Linkage analysis was performed using JoinMap® version 3.0 (Ooijen and Voorrips, 2002), employing the default settings. Linkage groups were determined using a LOD (logarithm of odds) threshold of 3.0, and genetic distances were computed using the Haldane mapping function. Genstat 16th edition was used for QTL detection and to estimate QTL effects using single marker analysis and the composite interval mapping (CIM) function.

### Identification and sequencing of candidate genes

Prior to the publication of the IWGSC RefSeq v1.0 genome assembly (Appels *et al.*, 2018), a variety of sources were used to identify candidate genes related to flowering or development within the QTL regions, from the available rice, *Brachypodium*, barley, and wheat sequences. Primers were designed to amplify the A-genome homoeologue of these candidates. PCR and sequencing reactions were carried out following the methods described by Zikhali *et al.* (2014). Newly discovered single nucleotide polymorphisms (SNPs) between Avalon and Cadenza were converted to KASP markers using PolyMarker (Ramirez-Gonzalez *et al.*, 2015) and validated in the BC_2_F_4_ recombinant lines. With the publication of the IWGSC RefSeq v1.0 genome assembly, it was possible to scrutinize all the genes covering the Ht and Hd QTLs from the sequence of Chinese Spring, with a region of 3AS between 45 Mb and 210 Mb analysed. In addition the RNAseq data (see below) were visualized in the Integrative Genomics Viewer (IGV, https://software.broadinstitute.org/software/igv) to identify SNPs. This approach identified an additional cohort of candidate genes from which new KASP markers were generated as above, where possible.

### RNAseq bulk segregant analysis strategy

Three recombinants from either the extreme early individuals (Avalon allele) or late flowering (Cadenza allele) individuals were combined into two bulks without replication. The bulks also carried the Avalon or Cadenza alleles, respectively, for height. Plants were vernalized for 8 weeks at 6 °C under SDs and then transferred to the glasshouse with a temperatur of ~18 °C and a LD photoperiod. The time point for sample collection, between the DR and TS, was selected based on the results of the developmental phase experiment.

Total RNA was prepared from the whole plant of each recombinant separately using the RNeasy^®^ Plant Mini Kit (Qiagen), followed by treatment with DNase I utilizing the RNase-Free DNase Set (Qiagen). RNA purification was performed using the RNeasy^®^ kit (Qiagen), according to the manufacturer’s protocol. Equivalent amounts of RNA from the three early or late recombinants were mixed to produce each RNA bulk sample.

Library construction and sequencing was performed by The Genome Analysis Centre (TGAC) in Norwich, UK. One Illumina TruSeq RNA version 2 library was constructed per bulk. Sequencing was carried out on the Illumina HiSeq2000 with 100 bp paired-end reads. The resulting reads were mapped to the wheat reference sequence using the RNAseq aligner STAR, with all default parameters chosen. The resulting BAM files were then processed with SAM tools and analysed using default parameters in Cufflinks, with reads being mapped to the reference sequence in FASTA format and reference annotation in GFF3 format. The Cufflinks output contained FPKM (fragments per kilobase of transcript per million mapped reads) values for each bulk sample. RPKM (reads per kilobase of transcript per million mapped reads) values were normalized by setting an RPKM cut-off in order to eliminate false discovery of high fold changes between genes with very low absolute expression levels (<0.1). Additionally, genes with an expression of 0 were rounded to a small number (0.001) to avoid logarithms of zero. Expression values were calculated using the formula log2fold change(Ava/Cad)=log2(FPKM_Ava)–log2(FPKM_Cad).

### Yield effects in selected 3A recombinants

Eight recombinant lines were used, together with parental varieties (Avalon and Cadenza) and NIL-A, and the lines were drilled in 6 m^2^ plots (after trimming), consisting of seven rows spaced 17 cm apart with a 60 cm path width, at a seed density of 250 m^–2^ in October 2015 (Morley Farm, Morley, Norfolk, 52°33′15.1′′N, 1°01′57.7′′E) and October 2016 (Church Farm, Bawburgh, Norfolk) in three replicates, with a fully randomized block design. The plants were grown according to standard agricultural practice, except that PGRs were not applied. The whole plots were harvested in August 2016 and 2017. GY was measured at the point of harvest by combine harvester. Thousand grain weight (TGW) was determined using a MARVIN Seed Analyser (GTA-Sensorik) and GN was calculated as grains m^–2^ by dividing GY by TGW. Crop height and heading date were scored as described above.

## Results

### Environmental sensitivity of 3A Hd and Ht QTLs

**Fig. 1.**
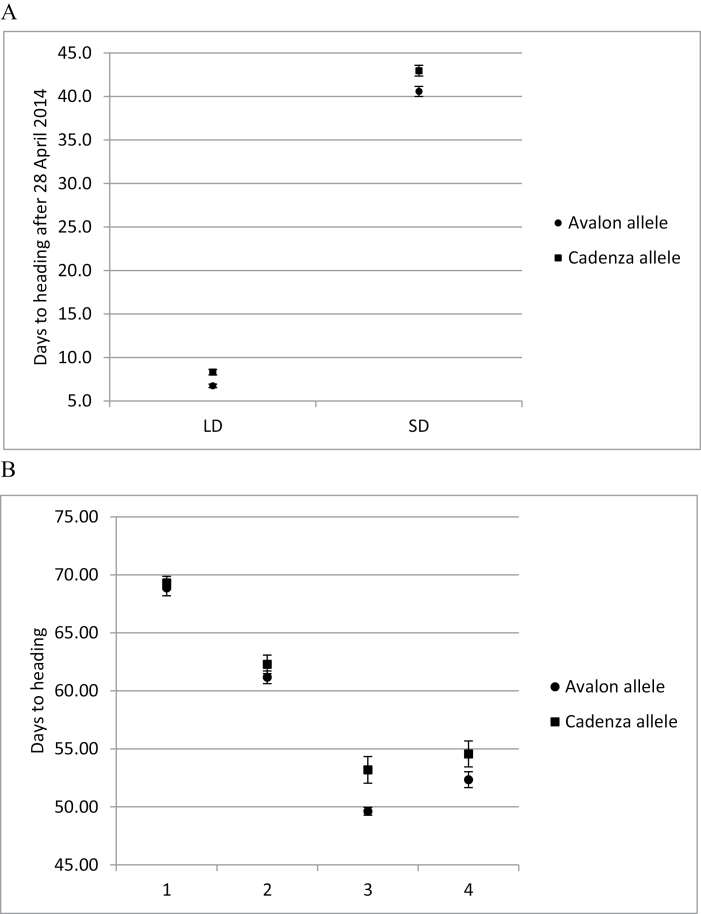
Average days to ear emergence (G55) for leading tillers of controlled-environment-grown Avalon×Cadenza NILs. Filled circles and filled squares represent the NILs carrying the Avalon and Cadenza alleles at 3A, respectively. (A) Short days (SDs) and long days (LDs) correspond to 10 h and 16 h light, respectively. (B) Response to vernalization using selected recombinant lines. Treatments for 0, 4, 6, and 8 weeks. The vertical bars indicate the SD.

The effect of the 3A Hd (Griffiths *et al.*, 2009), Ht (Griffiths *et al.*, 2012), and GY (Ma *et al.*, 2015) QTLs has been validated using the same isogenic materials under UK field conditions (Farré *et al.*, 2016). It is important to understand whether the 3A phenology effects are conditioned by sensitivity to photoperiod or vernalization. BC_2_ NILs segregating for the QTL region between *wmc505* and *wmc264* were assessed for Hd under controlled environments with fixed photoperiods and after a saturating vernalization treatment. In the photoperiod experiments, the Avalon 3A NILs headed significantly earlier then the Cadenza NILs (*P*<0.001) (1.58 d and 2.48 d under LD and SD conditions, respectively, Fig. 1A) without any significant interaction (*P*=0.321; interaction treatment×allele) which shows that the 3A heading QTL is not a photoperiod sensitivity (*Ppd*) effect.

To quantify the vernalization response, 32 out of the 76 recombinants used for fine mapping were selected to be grown in controlled environments (under LDs) after vernalization treatments of 0, 4, 6, or 8 weeks. The lines carrying the Avalon allele flowered earlier than those with the Cadenza allele for all vernalization treatments (Fig. 1B). Overall, a saturating vernalization treatment reduced heading date by ~2 weeks; Cadenza has a facultative growth habit and carries the dominant *Vrn-A1a* spring allele. Based on Wald testing, the allelic effect on heading date was highly significant (*P*<0.001) but there was no significant interaction between allele and vernalization treatment (*P*=0.192). However, the mean difference in heading date does increase with a longer duration of vernalization up to 6 weeks. From this we conclude that the 3A Hd QTL is not involved in vernalization sensitivity and, taken together with our day length response data, confirms the designation of the 3A Hd effect as earliness *per se* (*eps*).

### The 3A *eps* QTL affects the duration of early developmental phases

**Fig. 2.**
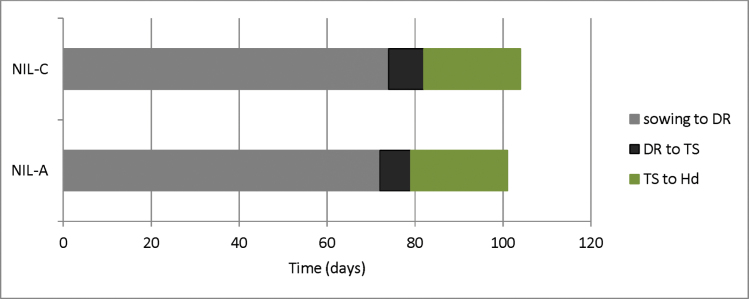
Duration of developmental phases of NIL-A (carrying the Avalon allele) and NIL-C (carrying the Cadenza allele). Developmental phases were divided into three phases: from sowing to double ridge (DR) in grey, from DR to terminal spikelet (TS) in black, and from TS to heading (Hd) in green. Differences between NIL-A and NIL-C were significant at *P*<0.001 for sowing to DR and DR to TS, but not for TS to Hd.

To determine which developmental phases were affected by the 3A *eps* gene, the time from sowing to heading was divided into three phases: from S to DR, from DR to TS, and from TS to Hd. NIL-A (carrying the Avalon allele) and NIL-C (carrying the Cadenza allele) are a pair of NILs from the AC179-E27-2 stream. The differences between them were 2 d and 1 d from S to DR and from DR to TS, respectively. This 3 d difference was maintained through to booting (*P*< 0.001) and Hd, so no differences were detected between the NILs in duration of the stem elongation period from TS to Hd (Fig. 2). These results indicate that the *eps* region affects the vegetative and early reproductive phases. Height and tiller number were also recorded in this experiment. NIL-C was 1.5 cm taller than NIL-A (*P*=0.049) and there was no significant difference in tiller number.

### Phenotype evaluation of recombinants

**Fig. 3.**
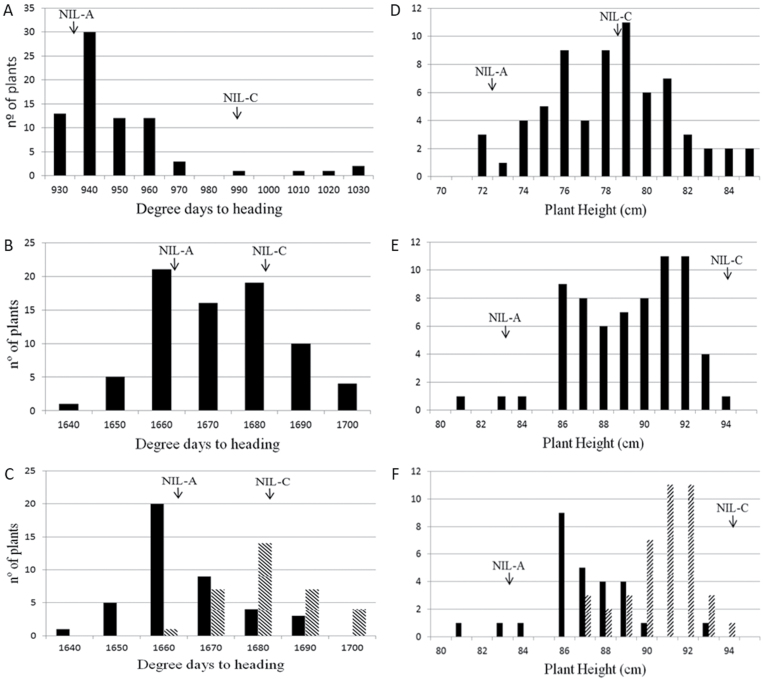
Phenotypic frequency distribution of degree days to heading and plant height in the BC_2_F_4_ population consisting of 76 spring-sown (A, D) and (B, E) autumn-sown lines. Arrows indicate means of degree days to heading and plant height for NIL-A and NIL-C. (C, F) Bars indicate lines with two genotype classes: homozygous for Avalon (black) and Cadenza (shaded) alleles according to the allele at the wmc505 and BS0003801 locus for Hd and Ht, respectively, using the autumn-sown data.

In order to compare the segregation of Ht and Hd at the 3A locus, 76 recombinant BC_2_F_4_ lines derived from crosses of NIL-A and NIL-C with Cadenza were phenotyped in two field experiments (one spring sown in 2013 and one autumn sown in 2013 and growing in 2013–2014; Fig. 3). For both sowing dates in natural field conditions, NIL-A (carrying the Avalon allele) flowered earlier than NIL-C (with Cadenza alleles) (NIL-A=938.78 and NIL-C=990.0 mean degree days to heading in the spring sowing and NIL-A=1663 and NIL-C=1681 in the autumn sowing). However, only the autumn-sown experiment showed a significant difference (*P*=0.017) in mean degree days to heading of the NILs for the 3AS QTLs. In contrast, the height difference between NILs was significant in all experiments.

### Mapping and QTL analysis

**Fig. 4.**
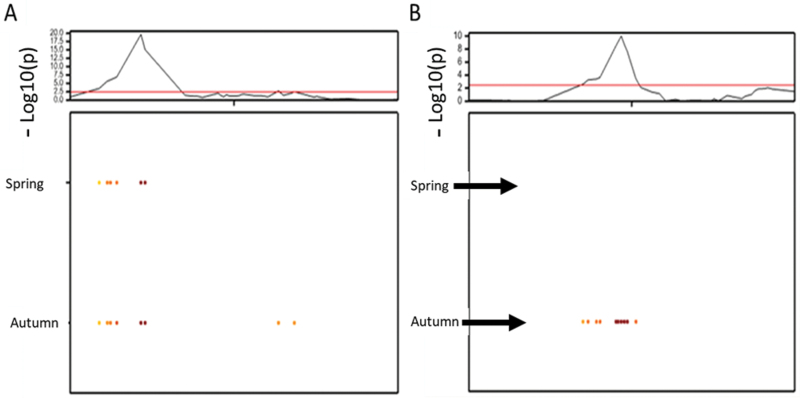
Presence of a QTL for Ht (A) and Hd (B) on chromosome 3A using the data from the 76 BC_2_F_4_ population for two trials, spring and autumn sown. The top panel shows the genome-wide profile; the black and red lines indicate the profile of –log10 (*P*-value) for a composite interval mapping scan. The red horizontal line shows the threshold value for significance (LOD=2.53). Below each graph is a representation of QTL additive effects detected from spring- and autumn-sown experiments. The darker orange/brown colours indicate an increasing additive effect with the Cadenza allele increasing for Ht and Hd.

**Fig 5.**
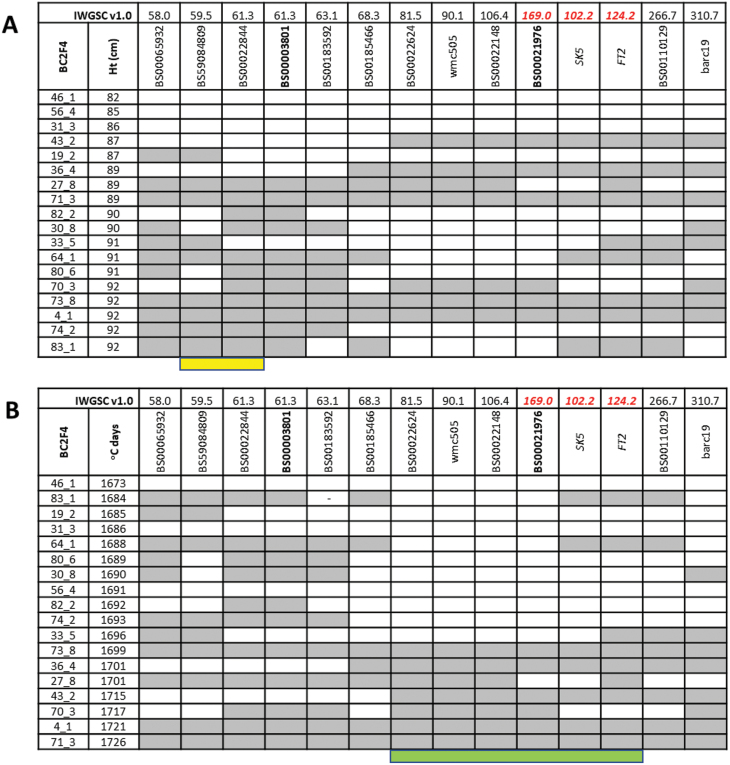
High-resolution genetic maps of the Ht and Hd QTL regions on chromosome 3A. The genotype for each marker and Ht or Hd data of 18 BC_2_F_4_ recombinants is shown. The top row of each figure shows the position of markers (second row) in the IWGSC RefSeq v1.0 (Mb). Positions in the RefSeq in red indicate a rearrngement relative to the Chinese Spring sequence. The peak QTL marker for each trait is shown in bold. Marker genotypes are shown in white (Avalon) or grey (Cadenza) in the recombinants. (A) The recombinants are ordered in increasing height (cm). Candidate genes for Ht would be in the region indicated in yellow. (B) The recombinants are ordered in increasing Hd (°C d). Candidate genes for Hd would be in the region indicated in green.

In order to refine the genetic interval containing the Hd, Ht, and ultimately GY effects, the NIL parents and 76 recombinant BC_2_F_4_ lines were screened using 65 KASP markers. These data were used to perform QTL analysis for the BC_2_F_4_ population for heading date and height to detect the QTLs. The results are presented in Fig. 4. Significant Hd and Ht QTLs were detected in the 3A region. The peak marker for Hd was BS00021976, which showed a positive effect for the Cadenza allele compared with the Avalon allele (additive effect=1.2 d); this was only observed in the autumn-sown trial. As expected from the NIL data, earlier flowering time is associated with the Avalon allele. The 3A Hd QTL contributed 36.15% of the phenotypic variation in heading date under field conditions. Ht is controlled by an independent QTL with the peak marker BS00022844 (which co-segregates with BS00003801, Fig. 5); again the allelic direction is the same as for the NILs with Cadenza increasing. Sorting a subset of the recombinants according to Ht (short to tall) or heading Hd (early to late) from the autumn-sown trial indicates the regions containing the Ht and Hd QTLs (Fig. 5). For Ht, the QTL region had been reduced to ~1.8 Mb (Fig. 5A); however, the Hd QTL could be in a region of at least 100 Mb, with the added complication of a likely rearrangement in that region (Fig. 5B).

### Examination of gene candidates

Publication of the IWGSC RefSeq v1.0 genome assembly from Chinese Spring (Appels *et al.*, 2018) allowed accurate positioning of the markers defining the QTL loci on Chr 3AS. The closest marker to the Ht QTL is BS00003801 at ~60 Mb in the IWGSC RefSeq v1.0, and that to the Hd QTL is BS00021976 (at ~169 Mb). The genomic sequences from the regions flanking these markers, ~5 Mb flanking BS00003801 and 60 Mb flanking BS00021976, were analysed for gene content. The sequences of the unspliced transcripts in these two regions were obtained using Biomart (EnsemblPlants) and then used to search the non-redundant protein sequences database for higher plants with blastx using the default parameters, at NCBI. This produced a list of 146 genes for Ht, and 536 genes for Hd, with ~75% assigned a gene identity. There are at least seven plausible gene candidates for each QTL, shown in Tables 1 and 2, based on gene function and/or expression level from the RNAseq analysis. Supplementary Table S1 lists the full high confidence gene content for the 265 Mb region covering the whole Ht and Hd QTL regions and was obtained using BioMart at https://plants.ensembl.org/Triticum_aestivum/Info/Index The Gene ID and the start and end positions in the IWGSC RefSeq v1.0 are indicated. Selected markers used in the QTL mapping (KASP and SSR) and the QTL regions they define for the Ht and Hd QTLs are shown. The log2 fold change between Cadenza and Avalon from the RNAseq data is also shown. Gene identity, if known, is indicated.

**Table 1.** List of candidate genes around the marker closest to the Ht QTL, BS00003801

Gene ID	Start	FPKM A	FPKM C	Log2fold change	ID	Function (if known)
Mapping marker						
TraesCS3A02G093400	59822956	0.09	0.04	1.33	Xyloglucan endotransglycosylase/hydrolase protein 8-like	Cell wall-modifying enzyme that can loosen cell walls leading to cell expansion and elongation
TraesCS3A02G093500	59954598	0.66	0.31	1.09	Xyloglucan endotransglycosylase/hydrolase protein 8-like	Cell wall-modifying enzyme that can loosen cell walls leading to cell expansion and elongation
TraesCS3A02G093600	60005281	1.12	0.82	0.45	Xyloglucan endotransglycosylase/hydrolase protein 8-like	Cell wall-modifying enzyme that can loosen cell walls leading to cell expansion and elongation
**TraesCS3A02G093700**	60028751	0.17	0.00	10.77	**Xyloglucan endotransglycosylase/hydrolase protein 8**	Cell wall-modifying enzyme that can loosen cell walls leading to cell expansion and elongation
**TraesCS3A02G093800**	60193220	0.00	0.01	–6.38	**Xyloglucan galactosyltransferase KATAMARI1 homologue**	Responsible for actin organization and the synthesis of cell wall materials
TraesCS3A02G094000	60203935	0.52	0.10	2.44	Glycine-rich cell wall structural protein-like	
**TraesCS3A02G094600**	60446831	0.89	0.55	0.69	**Cellulose synthase-like protein G2**	Cellulose biosynthesis and cell wall biogenesis
**QTL marker**	**61343069**				**BS00003801**	

Transcript name, start position in IWGSC v1.0 (Mb), FPKM values for the Avalon and Cadenza allele RNAseq bulks, the expression level ratio between Avalon and Cadenza (log2fold change), gene identity, and gene function, if known, are given. The significance level was defined as the log2fold change. Genes for which KASP markers have been developed are shown in bold.

**Table 2.** List of candidate genes and mapping markers around the marker closest to the Hd QTL, BS00021976

Gene ID	Start	FPKM A	FPKM C	Log2fold change	ID	Function (if known)
**Mapping marker**	81477543				**BS00022624**	
TraesCS3A02G116300	84184377	18.69	18.76	–0.01	*TaGI*	
**Mapping marker**					**wmc505**	
TraesCS3A02G122600	97972874	0.15	0.04	1.81	*Gibberellin 3-beta-dioxygenase 2-1*	
**Mapping marker**	106434896				**BS00022148**	
TraesCS3A02G133400	110332577	0.22	0.12	0.88	*Gibberellin 2-beta-dioxygenase 1-like*	
TraesCS3A02G136500	114041077	27.04	30.80	–0.19	*SHAGGY-like kinase*	
**TraesCS3A02G143100**	124172881	0.49	0.13	1.87	*FT2*	
TraesCS3A02G155200	147789276	54.43	60.82	–0.16	*IAA3*	
TraesCS3A02G156500	151642540	7.02	8.84	–0.33	*ABI8*	ABA response
TraesCS3A02G159200	158467595	0.49	0.65	–0.40	*ARF1*	Auxin response
**TraesCS3A02G164200**	168837089	27.17	31.41	–0.21	*TaSK5*	Brassinosteroid signalling pathway
***QTL Marker***	**168837302**				***BS00021976***	
TraesCS3A02G167100	172629517	2.77	3.20	–0.20		Putative brassinosteroid receptor
TraesCS3A02G173300	191527521	14.14	16.11	–0.19	*Topless*	
TraesCS3A02G176100	196761940	0.19	0.04	2.24	*FPF-L1*	
**Mapping marker**	266691232				**BS00110129**	

Transcript name, start position (in the IWGSC v1.0 sequence), FPKM values for the Avalon and Cadenza allele RNAseq bulks, the log2-transformed expression level ratio between Avalon and Cadenza bulks (log2fold change: positive values=Avalon expression higher, negative values=Cadenza expression higher), gene name, and gene function (if known) are shown. Genes for which KASP markers have been developed are shown in bold.

### Gene candidates for Ht

**Fig. 6.**
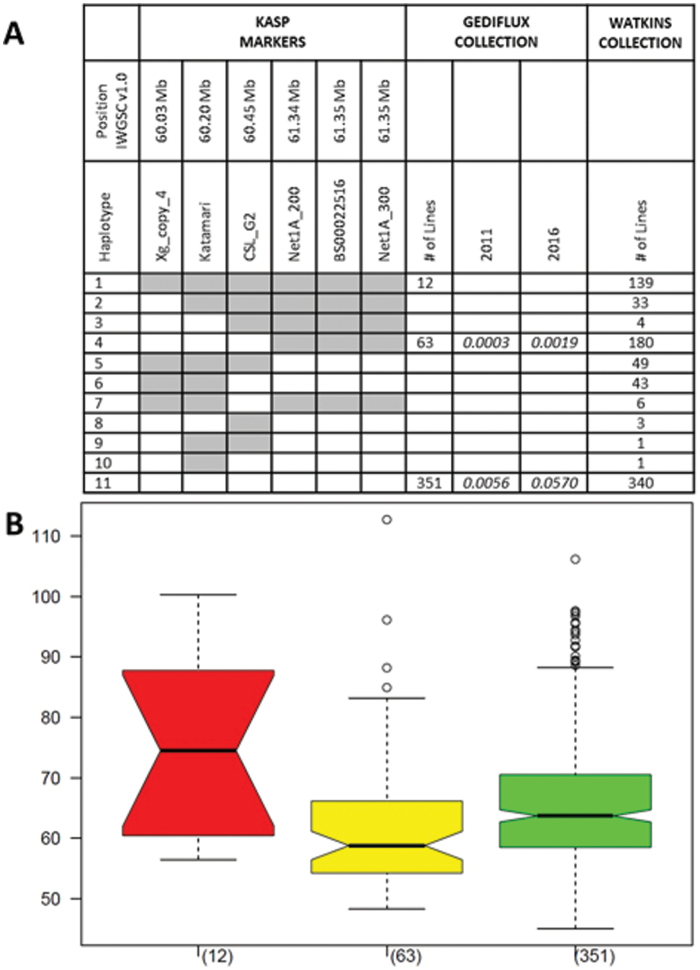
Haplotypes across the 3A Ht QTL locus in the Gediflux and Watkins collections. (A) Unshaded or shaded blocks represent the Avalon or Cadenza genotype for each marker in haplotypes 1–11. The position of the markers in the IWGSC Refseq is indicated. The significant differences between haplotype 1 and haplotypes 4 or 11 are shown for the Gediflux 2011 and 2016 trials. (B) Box plots indicating the Ht range (in cm) for each haplotype in the 2011 data. Red is haplotype 1, yellow haplotype 4, and green haplotype 11 (from A).

A cluster of genes involved in cell wall structure or synthesis, all of which could have a role in Ht, form an interesting group around the QTL peak marker (Table 1). The clustering could suggest a close interaction between some or all of the genes. There are four, almost identical and apparently functional copies of a xyloglucan endotransglycosylase/hydrolase protein 8 gene in Avalon and Cadenza, except for one probably non-functional copy in Avalon. Networked 1A appears to have been duplicated at this locus as the two transcripts are quite different. The first copy is very well conserved between Cadenza and Avalon, while the second copy shows a number of polymorphisms, which give both conservative and non-conservative amino acid changes in the Avalon protein compared with Cadenza and Chinese Spring. Another potential candidate is *cellulose synthase-like G2* (*CSL-G2*, which is involved in cellulose biosynthesis and cell wall biogenesis). *CSL-G2* has four SNPs between Avalon and Cadenza within exons, resulting in two amino acid changes. Transcriptomic analysis also shows expression level differences between some Avalon and Cadenza alleles in the cluster; most notably TraesCS3A02G093700, a xyloglucan endotransglycosylase, showing increased expression in lines carrying the Cadenza allele with a log2 fold change of >10 Supplementary Table S1 . KASP markers have been designed to SNPs within TraesCS3A01G093700.1 (Xg Copy 4), TraesCS3A01G093800.1 (Katamari), TraesCS3A02G094600 (CSL-G2), TraesCS3A01G096200.1 (Net1A_200), and TraesCS3A01G096300.1 (Net1A_300). These genes showed significant log2 fold change values and/or have gene functions with a possible effect on plant height. Along with BS00022516 (co-segregating with BS00003801), these markers have been tested on the Watkins (Watkins Stabilised Collection of Hexaploid Landrace Wheats) and Gediflux (Genetic Diversity Flux winter wheat collection) panels. The Gediflux panel gave three haplotypes in the region, only one of which shows recombination; this suggests that the locus has already been fixed in this geographically and temporally more limited collection (Fig. 6A). Analysis of Ht data from two trials of Gediflux (2011 and 2016) indicate that both the Avalon (haplotype 11) and recombinant (haplotype 4) haplotypes are significantly different from the Cadenza haplotype (haplotype 1), with the recombinant being the shorter (Fig. 6A, B). A comparison of haplotypes 4 and 11 shows that they are significantly different regarding plant height (2011: *P*=0.017, mean values 66.0 cm and 62.2 cm, respectively; 2016: *P*=0.0007, mean values 92.6 cm and 85.9 cm, respectively). The results suggest that the gene affecting Ht is distal to the marker Net1A_200 (21.34Mb); that is, xyloglucan endotransglycosylase/Katamari/cellulose synthase-like G2 or another unidentified gene, and confirms the position of the QTL from Fig. 5A.

The Watkins panel, a very diverse collection from across the globe, gave at least 11 haplotypes (Fig. 6A) apparently due to multiple recombinantion events. Due to the difficulty in collecting accurate height data in landraces and the probability of population structure, we cannot assign significance to the different haplotypes in Watkins. However, the results suggest that this small region (<1.5 Mb) may be a hotspot for recombination allowing different combinations of genes, which we suggest might have an effect on plant height, to come together. To examine this further, we analysed the Axiom genotyping data of 4 Mb over the cluster from a larger panel of mainly hexaploid varieties (including the Watkins and Gediflux lines). These data confirmed a high degree of recombination within the gene cluster (data not shown).

### Gene candidates for Hd

Prior to the publication of the IWGSC RefSeq v1.0 genome assembly from Chinese Spring (Appels *et al.*, 2018), several genes involved with flowering and development were identified in the wider genetic vicinity of the Hd QTL. These genes are found in pathways known to be involved in flowering time: photoperiod (*GI* and *CDF1*); and plant hormones, namely gibberellin (*GA2ox-5-3* and *FPF-L1*, *Flowering Promoting Factor 1-like 1*), auxin (*ARF1* and *IAA3*), and brassinosteroid signalling (*TaSK5*, a GSK3/SHAGGY-like kinase, and *BRI-associated receptor kinase*), and abscisic acid (*ABI8*). Publication of the IWGSC RefSeq v1.0 genome assembly allowed a closer examination of the genes around the marker at the Hd QTL peak. The marker with the highest association with the Hd QTL is KASP marker BS00021976. Table 2 shows possible gene candidates (based on gene function or expression differences) in the 60 Mb region either side of BS00021976, from its position in the CS sequence. We consider that *TaSK5*, *FT2* (*Flowering Locus T2*), or *FPF-L1* are the most promising gene candidates for Hd, based on likely gene function and expression differences. BS00021976 maps at 169 Mb in the IWGSC RefSeq v1.0 genome assembly to an SNP from Cadenza in the 3′-untranslated region (UTR) of TraesCS3A02G164200 (*TaSK5*). A KASP marker was developed to what appeared to be a second SNP in exon 4 of the same gene (in Avalon rather than Cadenza), causing an amino acid change. When this second marker was used for QTL mapping, it did not associate with Hd and was mapped genetically more distally on 3AS. Examination of the Chinese Spring sequence at this position (102 Mb) indicates the presence of an unannotated *GSK3/SHAGGY*-like kinase pseudogene, but we suggest that this sequence either must be incomplete or, more likely, that a duplicated copy of *TaSK5* is found at this location in Avalon as the CS sequences are not homologous enough to amplify the marker. In addition, there is another SHAGGY-like kinase at 114 Mb, which appears to be functional but again has insufficient homology to be amplified by the SNP2 marker. We therefore propose that the copy at 102 Mb has the exon 4 SNP, but is not involved in Hd, and the copy at 169 Mb, which has an SNP in the 3′-UTR, can be considered a candidate, although it shows little difference in expression level between Avalon and Cadenza. In addition, we suggest there has been a rearrangement around the Hd QTL as there is a divergence in the expected order of markers from the CS sequence when arranged according to genetic rather than physical distance (see Fig. 5). *FT2* is ~4.3 kb, consisting of four small exons. The sequence of the large intron 2 (2.9 kb) is incomplete. With the available data, three SNPs have been identified between Avalon and Cadenza, in the few transcripts from intron 2, but there are no polymorphisms in the coding regions. RNAseq data visualization in IGV indicates that most of exon 1 and all of exons 2 and 3 are missing in the Cadenza transcript at this developmental stage (Supplementary Fig. S2), which indicates that the complete *FT2* transcript is not expressed in Cadenza at the stage sampled here and therefore is unlikely to be functional. Expression of the gene is higher in Avalon, and higher expression of *FT2* has been shown to cause earlier flowering in Shaw *et al.* (2019).

*FPF1* does not show any polymorphisms between Avalon and Cadenza, but is more highly expressed in Avalon than in Cadenza. In Arabidopsis, *FPF1* is involved in transforming a vegetative meristem into an inflorescence meristem, and is therefore expressed in the equivalent developmental stages as the DR to TS growth stage in wheat (Melzer *et al*, 1999).

### Effect of 3A Ht and Hd loci on grain yield

**Fig. 7.**
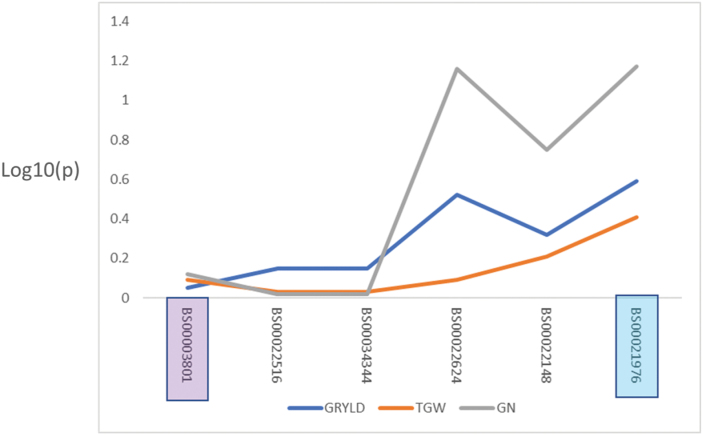
Single marker regression showing Log10(*P*) values from 2015–16 yield component data plotted in the marker order of the wheat genome reference sequence. The peak marker for Ht (BS00003801) is boxed in lilac and that for Hd (BS00021976) in blue. GY and GN are increasing effects for the Cadenza BS00021976 allele and reducing for TGW.

Eight informative recombinants (Supplementary Table S2A) were grown together with 3A NIL controls and the parents Avalon and Cadenza over two field seasons. As these lines were grown in relatively large (6 m^2^) plots, this provided the opportunity to dissect the locus in terms of GY. In the 2015–16 and 2016–17 growing seasons, the Ht and Hd results reported here (from the 1 m^2^ field experiments in 2013) and in Farre *et al.* (2016) were repeated. In 2016–17. Cadenza alleles at the 3A locus resulted in a crop which was later heading (by 1 d) and taller (11.7cm). The following season, the direction of allelic effect stayed the same for these two traits but was reduced to 0.67 d for Hd and to 10.2 cm for Ht. For GY, the Cadenza allele conferred an increase in 2015–16 (Supplementary Table S2A). The GY of NIL-C and NIL-A was 10.65 t ha^–1^ and 9.52 t ha^–1^, respectively (*P*=0.07). The NIL difference in GY was driven by grains per unit area (GRpsqm) which was 21 264 compared with 19 192. This repeated the findings of Farre *et al.* (2016), but there were no GY difference between the NILs for 2016–17; for this reason, the effect of the locus on GY in recombinant lines was analysed using only the 2015–16 data (shown in Supplementary Table S2B). In Fig. 7*P*-values of each of the markers spanning the locus are shown for GY, TGW, and GN (the single marker regression is shown in Supplementary Table S2B). None of these differences show *P*-values <0.05. However, a trend of increasing additive effect associated with Cadenza alleles (not shown) and decreasing *P*-value is seen for markers closer to the Hd locus. Single marker regression showed that *XBS00021976* was most significantly associated with grains per unit area (*P*-value 0.07) and GY (*P*-value 0.26).

## Discussion

In the grasses, stem extension and reproductive development (and so the formation of yield components) are very tightly linked developmental processes. Stem extension begins once the inflorescence has reached the terminal spikelet and ends around the same time as anthesis, so there is correlation between traits associated with each process. This correlation can be seen at the molecular level, with the same gene affecting stem extension, phenology, and GY; for example, *Ghd8* in rice (Yan *et al.*, 2011). However, we show that the Ht and Hd QTLs co-located on chromosome 3A (Griffiths *et al.*, 2009, 2012; Ma *et al.*, 2015) are genetically linked but independent effects (Fig. 4). We have used genetic recombination to separate them, effectively developing sets of sub-NILs which display height and heading differences in isolation (Fig. 5). This opens the way for the independent selection of these loci in breeding. Analysis of the haplotypes across the Ht locus within the AE Watkins landrace collection and the Gediflux collection of 20th Century European winter wheat shows that historical recombinants of this kind have occurred multiple times, with 11 haplotypes apparent (Fig. 6). The genetic separation of Ht and Hd also allowed us to ask how each of them was contributing to the GY QTL which we had previously shown to co-locate on chromosome 3A. The answer to this question has important implications for the value of the yield effect. If the GY effect was simply due to greater height and more light interception by taller lines in plots, the results might not be transferable to farmers, but our data show that GY is not a pleiotropic effect of the Ht locus. The proposal that the GY- and Hd-increasing alleles of Cadenza belong to the same gene is plausible (Fig. 7).

### Gene candidates for Ht, Hd, and GY

The Hd interval spans the centromere of chromosome 3A over a relatively large physical distance. The equivalent region of Chinese Spring is 229 Mb and contains 536 genes. This part of the 3A locus was intractable to further genetic resolution in this work due to reduced levels of recombination encountered in centromeric regions. The 3A Ht effect is more distally located on 3AS in a 5 Mb equivalent region of Chinese Spring containing 146 genes. In spite of these large gene numbers, analysis of known function, polymorphism between Avalon and Cadenza, and expression level differences did support the proposal of gene candidates for the 3A QTL complex that we had set out to dissect.

For Ht, the cluster of cell wall-related genes and expression level differences from them could be resolved to some extent using the historical recombination events present in the Gediflux collection (Fig. 6). This pointed towards the *Xyloglucan endotransglycosylase*, Katamari, and *cellulose synthase-like G2* part of the cluster being prioritized as most probably containing the causative gene(s). Xyloglucan is an essential component in the formation and function of the plant cell walls, and both the xyloglucan endotransglycosylases and Katamari (MUR3) are involved in these processes. Xyloglucan endotransglucosylases/hydrolases catalyse the endo cleavage of xyloglucan polymers and appear to have a role in cell wall restructuring. The rice orthologue, *OsXTH8*, is thought to be involved in this process and is regulated by GA, with increased expression leading to increased plant height (Jan *et al.*, 2004). Plants synthesize xyloglucan which contain galactose in two types of side chain. In Arabidopsis, mutants of MUR3 missing one type of side chain have a dwarf phenotype (Kong *et al.*, 2015). Cellulose is the most important component of plant cell walls, required to maintain shape and rigidity. Cellulose synthase-like genes may be involved in the synthesis of the backbones of hemicelluloses In rice, DNL1 is a major QTL for plant height and encodes OsCSLD4 (Ding *et al*., 2015). An unidentified glycine-rich cell wall structural protein-like is also located within this region.

For Hd and GY, it is *FT2* that stands out from the list of candidates (Table 2) as the best in terms of its known dual effects on Hd and spike fertility. As reported by Shaw *et al.* (2019) in durum wheat, *FT2* late alleles also confer increased spikelet number, which was also observed by Farré *et al.* (2016) in the same material as described here. In addition, Shaw *et al.* (2019) observed floret number per spikelet increases also observed by Ochagavía *et al.* (2018) in the Avalon/Cadenza NILs. In Shaw *et al.* (2019), the observed fertility changes were not accompanied by a yield increase but they do provide a physiological footprint, beyond simple phenology, analogous to spike fertility, and GY effects reported here and in our previous work. For example, Farré *et al.* (2016) reported a significant increase in grains per spike and spikelets per spike conferred by the Cadenza 3A allele studied here; there was no significant effect of spikes per unit area.

### Further delimitation of the GY QTL

Most of the Ht and Hd work described here was done in small plots which are not appropriate for yield estimation. However, informative recombinants were grown in large (6 m^2^) replicated plots to better understand how the independent Ht and Hd effects influence GY. This experiment did not identify significant GY differences but did show a clear trend for a yield difference, equivalent to that seen between NILs, in recombinants differing for the Hd QTL but the same mean GY for Cadenza and Avalon alleles at the Ht QTL (Fig. 7). The evidence presented here supports the location of the GY effect as within the newly defined Hd locus. It is interesting to note that in the two seasons of yield trial described here, the year when the NIL parents did not significantly differ in Hd they also did not differ in GY, while the Ht phenotype was strongly expressed. As already stated, FT2 is a candidate for the Hd and GY QTL. The work presented here supports the value of further exploration of the role of FT2 in spike fertility and GY.

### Environmental sensitivity of Hd effect

It is important to understand how the 3A Hd effect interacts with environmental signals, not only for its role in adaptation but also for our speculation that the same gene is influencing GY. Controlled-environment studies showed that this is not a new *Vrn* or *Ppd* gene. In fact, it fulfils the classical categorization as earliness *per se*, with a difference in Hd still evident after saturating vernalization and photoperiod (Fig. 1). It is also clear that the *eps* effect acts on the vegetative to floral transition. This QTL does exhibit interesting genotype by environment interactions. We showed that, when expressed, the developmental difference is apparent at the vegetative and early reproductive phases, with no difference in the duration of the late reproductive phase (Fig. 2). The Hd effect was not expressed after spring drilling. In the vernalization experiments, the additive effect increased when vernalization was fully satisfied. Elsewhere, Ochagavia *et al.* (2018) demonstrated that the NILs did not show any phenology differences in Northern Spain. In experiments not presented here, we also showed no significant effect on phenology in 16 °C and 21 °C controlled-environment experiments. Taken together, these data point towards a possible role for ambient temperature sensitivity differences, during vegetative growth, between Avalon and Cadenza alleles. Previous studies challenged the idea of earliness *per se* as a wholly autonomous developmental process occurring without environmental interaction. Indeed, we showed that *EPS-D1* exhibits a strong interaction with temperature (Ochagavía *et al.*, 2019), and it seems likely that this is also the case for the 3A Hd effect described here, that should probably be named *EPS-A2*.

## Supplementary data

The following supplementary data are available at *JXB* online.

Fig. S1. Meteorological data relating to field experimentation.

Fig. S2. Screen shots of FT2 from IGV.

Table S1. Expression level differences for predicted genes of the whole 3A locus.

Table S2. GY data and single marker regression.

erab058_suppl_Supplementary-Figures-S1-S2_Table-S1-S2

## Data Availability

All data used in this study are included in the manuscript figures and tables or in the supplementary data. All germplasm is available from SG.

## Abbreviations

DR: double ridge
eps: earliness *per se*
NIL: near isogenic line
Ppd: photoperiod
QTL: quantitative trait locus
Rht: reduced height
RPKM: reads per kilobase of transcript per million mapped reads
S: sowing
TS: terminal spikelet
Vrn: vernalization.
