# Supplementary material for: Resolving a QTL complex for height, heading, and grain yield on chromosome 3A in bread wheat
Source: J Exp Bot. 2021 Feb 12;72(8):2965–78. doi: 10.1093/jxb/erab058 (PMC8023219; doi:10.1093/jxb/erab058)
Supplement: erab058_suppl_Supplementary-Figures-S1-S2_Table-S1-S2 [file erab058_suppl_supplementary-figures-s1-s2_table-s1-s2.pdf]

Resolving a QTL complex for height, heading, and grain yield on chromosome 3A in bread wheat

Alba Farre Martinez, Clare Lister, Sue Freeman, Jun Ma, Simon Berry, Luzie Wingen, Simon Griffiths

Supplementary Fig 1

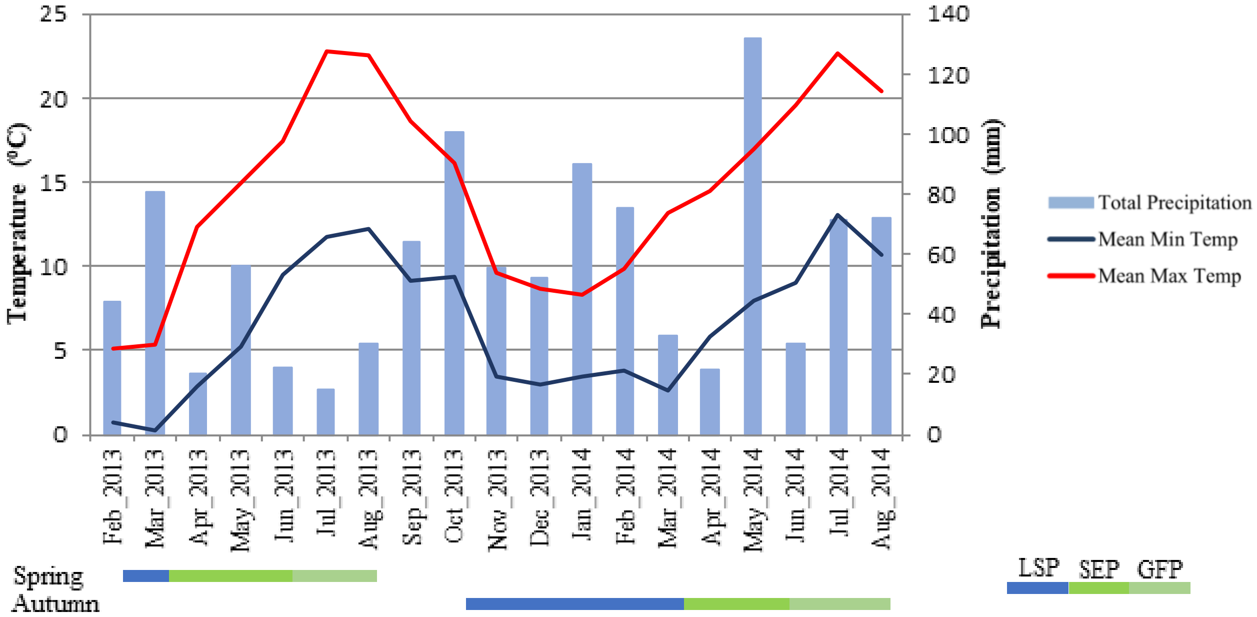

Supplementary Fig 1. Means of minimum (blue line) and maximum (red line) temperatures and cumulated rainfall (blue bars) during the months of the growth cycle of the spring and autumn sown trials (LSP = period from seedling emergence to first node detectable, SEP = period from first node detectable to anthesis and GFP = period from anthesis to physiological maturity).

Supplementary Fig 2

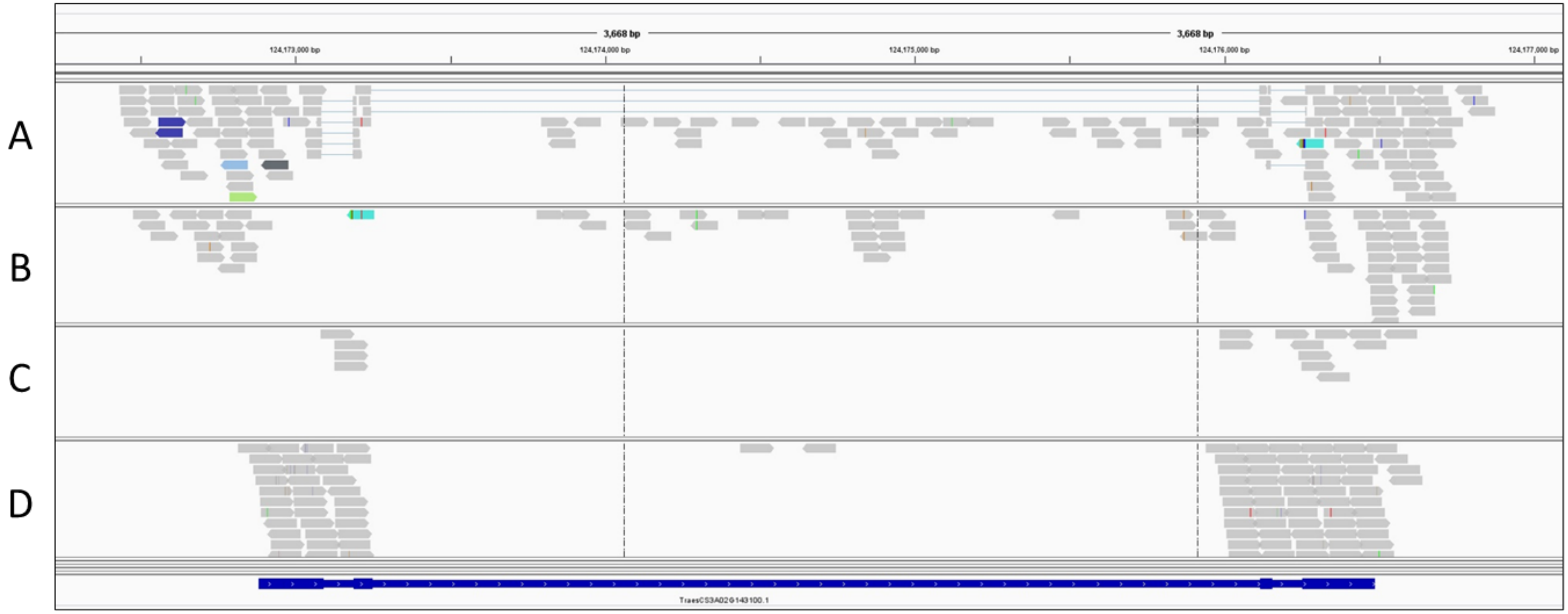

Supplementary Fig. 2

Screenshot from IGV showing expression of *FT2* = TraesCS3A02G143000

A. RNAseq data from the Early/Short pools, equivalent to Avalon
B. RNAseq data from the Late/Tall pools, equivalent to Cadenza
C. Exome capture data from Avalon
D. Exome capture data from Cadenza

Supplementary Table 1

Gene ID	Start	QTL Region	Mapping Marker	log2 FoldChange	Gene
TraesCS3A02G072900	45083541			-0.09	
TraesCS3A02G073000	45124474			0.30	
TraesCS3A02G073100	45170467			0.00	
TraesCS3A02G073200	45564043			0.50	
TraesCS3A02G073300	45569687			-0.22	
TraesCS3A02G073400	45592602			-0.20	
TraesCS3A02G073500	45776166			0.00	
TraesCS3A02G073600	45931648			-1.27	
TraesCS3A02G073700	46006549			0.04	
TraesCS3A02G073800	46022717			0.06	
TraesCS3A02G073900	46067850			0.00	
TraesCS3A02G074000	46164880			-0.27	cleavage stimulation factor subunit 77
TraesCS3A02G074100	46211647			0.00	
TraesCS3A02G074200	46457067			0.00	
TraesCS3A02G074300	46565724			1.33	
TraesCS3A02G074400	46574627			0.71	
TraesCS3A02G074500	46582312			-0.26	
TraesCS3A02G074600	46604044			0.11	
TraesCS3A02G074700	46610924			0.00	
TraesCS3A02G074800	46713189			0.15	
TraesCS3A02G074900	46729244			-0.24	
TraesCS3A02G075000	46829588			-0.29	
TraesCS3A02G075100	46833853			-0.09	
TraesCS3A02G075200	46844208			0.00	
TraesCS3A02G075300	46867165			0.00	
TraesCS3A02G075400	46870278			-0.15	
TraesCS3A02G075500	46985549			-0.19	
TraesCS3A02G075600	46992770			0.03	

TraesCS3A02G075700	47036289			-0.33	
TraesCS3A02G075800	47046436			-0.09	
TraesCS3A02G075900	47047692			1.06	
TraesCS3A02G076000	47063604			-0.28	putative disease resistance RPP13-like protein 3
TraesCS3A02G076100	47065853			0.36	
TraesCS3A02G076200	47083095			0.61	
TraesCS3A02G076300	47246260			-0.71	
TraesCS3A02G076400	47260433			0.01	
TraesCS3A02G076500	47277052			1.26	
TraesCS3A02G076600	47283507			7.48	BTB/POZ and MATH domain-containing protein 2-like
TraesCS3A02G076700	47491958			0.00	
TraesCS3A02G076800	47524317			0.00	
TraesCS3A02G076900	47821346			-0.32	
TraesCS3A02G077000	47825261			0.11	
TraesCS3A02G077100	47847824			-1.86	
TraesCS3A02G077300	48663110			3.19	Premnaspirodiene oxygenase
TraesCS3A02G077400	49478053			-0.16	
TraesCS3A02G077500	49504523			-0.14	
TraesCS3A02G077600	49973825			-0.13	
TraesCS3A02G077700	49980885			-0.18	
TraesCS3A02G077800	50365107			1.04	
TraesCS3A02G077900	50382036			0.00	
TraesCS3A02G078000	50436222			0.00	
TraesCS3A02G078100	50546343			-0.32	ricin B-like lectin R40G3
TraesCS3A02G078200	50638990			0.09	
TraesCS3A02G078300	50720217			0.22	
TraesCS3A02G078400	50726169			0.51	NAC domain-containing protein 29
TraesCS3A02G078500	50747138			-0.16	
TraesCS3A02G078600	50818561			-0.37	Endosomal targeting BRO1-like domain-containing protein
TraesCS3A02G078700	50888369			-0.26	
TraesCS3A02G078800	50889917			-0.42	
TraesCS3A02G078900	50896552			-0.30	

TraesCS3A02G079000	50936049			0.00	
TraesCS3A02G079100	50951929			0.00	
TraesCS3A02G079200	51280020			-0.27	
TraesCS3A02G079300	51381392			2.06	F-box protein PP2-B10-like
TraesCS3A02G079400	51401167			-0.04	
TraesCS3A02G079500	51408022			7.42	unknown
TraesCS3A02G079700	51696478			0.00	
TraesCS3A02G079800	51750498			0.00	Calmodulin-binding transcription activator 2
TraesCS3A02G079900	51768701			-0.20	
TraesCS3A02G080000	51810649			0.00	
TraesCS3A02G080100	51868745			2.45	maturase, partial (mitochondrion)
TraesCS3A02G080200	51870813			4.60	uncharacterized mitochondrial protein
TraesCS3A02G080300	51916014			0.00	
TraesCS3A02G080400	51917341			1.98	Cytochrome c oxidase subunit 1
TraesCS3A02G080500	51946648			-6.68	unknown
TraesCS3A02G080600	52207283			1.92	protein PHLOEM PROTEIN 2-LIKE A10-like
TraesCS3A02G080700	52347273			-0.32	
TraesCS3A02G080800	52375618			0.50	
TraesCS3A02G080900	52418687			-0.08	
TraesCS3A02G081000	52446596			0.00	
TraesCS3A02G081100	52457399			1.46	protein NRT1/ PTR FAMILY 6.1
TraesCS3A02G081200	52548451			0.00	
TraesCS3A02G081300	52555769			1.39	light-regulated protein [Aegilops tauschii subsp. tauschii]
TraesCS3A02G081400	52556940			0.04	
TraesCS3A02G081500	52589221			0.00	
TraesCS3A02G081600	52680461			0.00	
TraesCS3A02G081700	52726292			0.00	
TraesCS3A02G081800	53035863			-0.21	
TraesCS3A02G081900	53182880			-0.29	
TraesCS3A02G082000	53203757			-0.09	
TraesCS3A02G082100	53209389			0.15	
TraesCS3A02G082200	53215407			-6.28	cysteine-rich repeat secretory protein 2-like

TraesCS3A02G082300	53217862			0.02	
TraesCS3A02G082400	53256653			-8.03	NDR1/HIN1-like protein 3
TraesCS3A02G082500	53293163			0.00	
TraesCS3A02G082600	53323291			0.00	
TraesCS3A02G082700	53332151			-0.03	
TraesCS3A02G082800	53342113			-0.13	
TraesCS3A02G082900	53414134			6.45	cytochrome P450 704C1-like
TraesCS3A02G083000	53553821			0.27	
TraesCS3A02G083200	53559328			0.54	non-specific phospholipase C6-like
TraesCS3A02G083300	53566802			-0.28	
TraesCS3A02G083400	53568768			1.66	unknown
TraesCS3A02G083500	53569247			-0.24	
TraesCS3A02G083600	53738600			0.50	
TraesCS3A02G083700	53832244			0.32	
TraesCS3A02G083800	53838186			-0.19	
TraesCS3A02G083900	54232013			0.00	
TraesCS3A02G084000	54283987			0.00	
TraesCS3A02G084100	54325846			1.74	transmembrane protein 45B-like
TraesCS3A02G084200	54466317			0.00	
TraesCS3A02G084300	54655350			-0.25	
TraesCS3A02G084400	54658038			-0.09	
TraesCS3A02G084500	54661648			-0.04	
TraesCS3A02G084600	54666578			-0.29	
TraesCS3A02G084700	54804477			7.46	cytochrome P450 704C1-like
TraesCS3A02G084800	54874740			0.25	
TraesCS3A02G084900	54878375			0.24	
TraesCS3A02G085000	54883449			-1.14	putative pentatricopeptide repeat-containing protein At2g01510
TraesCS3A02G085200	54938812			0.01	
TraesCS3A02G085300	55031174			-0.11	
TraesCS3A02G085400	55035634			-0.14	
TraesCS3A02G085500	55045418			-0.32	
TraesCS3A02G085600	55085818			-0.06	

TraesCS3A02G085700	55223622			0.64	
TraesCS3A02G085800	55483864			0.00	
TraesCS3A02G085900	55501921			0.33	
TraesCS3A02G086000	55558213			0.00	
TraesCS3A02G086100	55705926			-0.99	Cytochrome P450 99A2
TraesCS3A02G086200	55716008			-0.16	
TraesCS3A02G086300	55722831			-6.23	probable disease resistance protein At1g61300
TraesCS3A02G086400	55737602			-0.24	
TraesCS3A02G086500	55743110			-7.36	unknown
TraesCS3A02G086600	55841050			0.00	
TraesCS3A02G086700	56133676			-0.17	Glyoxysomal fatty acid beta-oxidation multifunctional protein MFP-a [Triticum urartu]
TraesCS3A02G086800	56141475			-0.12	
TraesCS3A02G086900	56215736			0.21	DNA-damage-repair/toleration protein DRT111, chloroplastic [Aegilops tauschii subsp. tauschii]
TraesCS3A02G087000	56219767			-0.32	Cysteine synthase, chloroplastic/chromoplastic [Triticum urartu]
TraesCS3A02G087100	56226520			-0.27	cysteine synthase-like
TraesCS3A02G087200	56389147			0.33	ethylene-responsive transcription factor ERF056-like [Aegilops tauschii subsp. tauschii]
TraesCS3A02G087300	56390066			1.44	putative NADP-dependent oxidoreductase P1
TraesCS3A02G087400	56395506			-0.13	putative uridine nucleosidase 2 [Zea mays]
TraesCS3A02G087500	56397840			-0.11	RAP domain-containing protein, chloroplastic [Aegilops tauschii subsp. tauschii]
TraesCS3A02G087600	56409237	HT		0.00	putative F-box/LRR-repeat protein At3g28410 [Aegilops tauschii subsp. tauschii]
TraesCS3A02G087700	56590829			1.13	GDSL esterase/lipase At1g33811-like
TraesCS3A02G087800	56668613			-0.33	transcription factor GTE8-like [Aegilops tauschii subsp. tauschii]
TraesCS3A02G087900	56734766			8.77	RING-H2 finger protein ATL5-like
TraesCS3A02G088000	56762904			-0.82	probable LRR receptor-like serine/threonine-protein kinase At3g47570 [Aegilops tauschii subsp. tauschii]
TraesCS3A02G088100	56765030			0.91	probable LRR receptor-like serine/threonine-protein kinase At3g47570 [Aegilops tauschii subsp. tauschii]
TraesCS3A02G088200	56776222			0.00	Subtilisin inhibitor 1 [Triticum urartu]
TraesCS3A02G088300	56778942			0.00	Subtilisin inhibitor 1 [Triticum urartu]
TraesCS3A02G088400	56850923			0.00	Subtilisin inhibitor 1 [Triticum urartu]
TraesCS3A02G088500	56853792			0.00	Subtilisin inhibitor 1 [Triticum urartu]

TraesCS3A02G088600	56858691			0.00	Subtilisin inhibitor 1 [Triticum urartu]
TraesCS3A02G088700	56948633			0.00	Subtilisin inhibitor 1 [Triticum urartu]
TraesCS3A02G088800	57013076			0.53	histone H2B.1-like [Aegilops tauschii subsp. tauschii]
TraesCS3A02G088900	57017091			-0.34	Transpososon
TraesCS3A02G089000	57090614			-0.21	Aspartyl-tRNA synthetase [Triticum urartu]
TraesCS3A02G089100	57154066			-7.73	
TraesCS3A02G089200	57158330			-0.30	protein BREAST CANCER SUSCEPTIBILITY 2 homolog B isoform X6
TraesCS3A02G089300	57195259			-7.23	protein trichome birefringence-like 18
TraesCS3A02G089400	57215581			0.00	
TraesCS3A02G089500	57226198			0.00	
TraesCS3A02G089600	57243781			0.00	
TraesCS3A02G089700	57260777			-0.38	Dol-P-Man:Man(5)GlcNAc(2)-PP-Dol alpha-13-mannosyltransferase [Zea mays]
TraesCS3A02G089900	57276120			0.00	
TraesCS3A02G090000	57277812			-0.20	Transpososon
TraesCS3A02G090100	57374570			-0.11	Beta-hexosaminidase 3 [Hordeum vulgare]
TraesCS3A02G090300	57481565			0.00	MADS-box transcription factor 29-like [Aegilops tauschii subsp. tauschii]
TraesCS3A02G090400	57514756			0.00	chaperone protein ClpB1-like isoform X1 [Aegilops tauschii subsp. tauschii]
TraesCS3A02G090500	57560501			-0.48	zinc finger MYM-type protein 1-like, partial [Panicum hallii]
TraesCS3A02G090700	57611063			0.00	
TraesCS3A02G090800	57702664			-0.21	
TraesCS3A02G090900	57715937			0.00	MADS-box transcription factor 29-like [Aegilops tauschii subsp. tauschii]
TraesCS3A02G091000	57774541			0.45	STIP1-like protein
TraesCS3A02G091100	57804104			-0.13	Protein NSP-INTERACTING KINASE 3
TraesCS3A02G091200	57812878			3.04	
TraesCS3A02G091400	57855684		**BS00013584**	0.10	SH3 domain-containing protein
TraesCS3A02G091500	57859957			0.00	Desiccation protectant protein Lea14-like protein
TraesCS3A02G091600	57864567			-0.22	tRNA pseudouridine(38/39) synthase isoform X2
TraesCS3A02G091700	57873409			0.00	
TraesCS3A02G091800	57887384			-0.54	G-type lectin S-receptor-like serine/threonine-protein kinase SD2-5 [Hordeum vulgare]
TraesCS3A02G091900	57894487			-2.89	pentatricopeptide repeat-containing protein At1g66345, mitochondrial

TraesCS3A02G092000	57896894			0.14	universal stress protein PHOS32-like [Aegilops tauschii subsp. tauschii]
TraesCS3A02G092100	57898825			-0.83	
TraesCS3A02G092200	57903709			-1.09	F-box/LRR-repeat protein 14-like
TraesCS3A02G092300	57943487			1.25	probable LRR receptor-like serine/threonine-protein kinase At4g26540
TraesCS3A02G092400	57958367			-0.43	RNA-binding protein 10 isoform X3
	58045380		BS00065932		
TraesCS3A02G092500	58077003			0.00	
TraesCS3A02G092600	58467981			0.00	
TraesCS3A02G092700	58683126			0.91	putative ZmEBE-1 protein
TraesCS3A02G092800	58836094			2.04	galactinol--sucrose galactosyltransferase
TraesCS3A02G092900	58927199			-0.21	PREDICTED: vegetative cell wall protein gp1-like [Oryza brachyantha]
TraesCS3A02G093000	59424278			0.27	tryptophan aminotransferase related 2
TraesCS3A02G093100	59508291			-0.16	protein RST1 isoform X1
	59521691		BS59084809i		
TraesCS3A02G093200	59531321			1.54	protein LATERAL ORGAN BOUNDARIES-like
TraesCS3A02G093300	59806641			-0.44	zinc finger protein 511
TraesCS3A02G093400	59822956			1.33	xyloglucan endotransglycosylase/hydrolase protein 8-like
TraesCS3A02G093500	59954598			1.09	xyloglucan endotransglycosylase/hydrolase protein 8-like
TraesCS3A02G093600	60005281			0.45	xyloglucan endotransglycosylase/hydrolase protein 8-like
TraesCS3A02G093700	60028751			10.77	Xyloglucan endotransglycosylase/hydrolase protein 8 [Triticum urartu]
TraesCS3A02G093800	60193220			-6.38	60.2 Mb xyloglucan galactosyltransferase KATAMARI1 homolog [Aegilops tauschii subsp. tauschii]
TraesCS3A02G093900	60200264			-0.24	Subtilisin-chymotrypsin inhibitor-2A [Triticum urartu]
TraesCS3A02G094000	60203935			2.44	glycine-rich cell wall structural protein-like
TraesCS3A02G094100	60242518			-7.44	
TraesCS3A02G094200	60250917			0.00	
TraesCS3A02G094300	60297385			0.00	myb-related protein 305-like [Aegilops tauschii subsp. tauschii]
TraesCS3A02G094400	60434066			3.05	
TraesCS3A02G094500	60441183			0.00	premnaspirodiene oxygenase-like [Aegilops tauschii subsp. tauschii]
TraesCS3A02G094600	60446831			0.69	Cellulose synthase-like protein G2 [Triticum urartu]
TraesCS3A02G094700	60471506			0.00	Premnaspirodiene oxygenase [Triticum urartu]

TraesCS3A02G094800	60501708			0.00	U-box domain-containing protein 12-like [Aegilops tauschii subsp. tauschii]
TraesCS3A02G094900	60504891			1.18	
TraesCS3A02G095000	60506864			0.09	probable leucine-rich repeat receptor-like protein kinase At5g49770 [Aegilops tauschii subsp. tauschii]
TraesCS3A02G095100	60611287			0.56	dihydrolipoyllysine-residue acetyltransferase component of acetoin cleaving system-like [Hordeum vulgare]
TraesCS3A02G095200	60629607			-0.04	Peptide-O-fucosyltransferase [Zostera marina]
TraesCS3A02G095300	60663849			-0.09	WD repeat domain phosphoinositide-interacting protein 3 [Hordeum vulgare]
TraesCS3A02G095400	60682662			-0.09	RING finger and transmembrane domain-containing protein 2-like [Hordeum vulgare]
TraesCS3A02G095600	60842400			0.00	dimeric alpha-amylase inhibitor
TraesCS3A02G095700	60867810			0.00	putative disease resistance RPP13-like protein 3 [Aegilops tauschii subsp. tauschii]
TraesCS3A02G095800	61213997			-0.07	
	61219539		**BS00034344**		
	61308017		BS00022844		
TraesCS3A02G095900	61308087			1.75	
TraesCS3A02G096000	61311578			-1.37	amino-acid permease BAT1 homolog
TraesCS3A02G096100	61317610			0.21	putative F-box protein At1g47765
TraesCS3A02G096200	61340198		BS00003801	0.07	protein NETWORKED 1A [Aegilops tauschii subsp. tauschii]
TraesCS3A02G096300	61346177		BS00022516 BS00013997	-0.04	protein NETWORKED 1A [Aegilops tauschii subsp. tauschii]
TraesCS3A02G096400	61437310			0.00	
TraesCS3A02G096500	61445227			-0.64	cysteine-rich receptor-like protein kinase 2 isoform X2 [Aegilops tauschii subsp. tauschii]
TraesCS3A02G096600	61478511			1.18	serine/threonine-protein phosphatase 7 long form homolog
TraesCS3A02G096700	61482512			2.70	pentatricopeptide repeat-containing protein At5g66631
TraesCS3A02G096800	62421465			-0.07	
TraesCS3A02G096900	62433938			0.91	
TraesCS3A02G097000	62445661			-0.12	
TraesCS3A02G097100	63065555			-0.07	Nucleotide/sugar transporter family protein [Zea mays]
TraesCS3A02G097200	63071948			-0.45	mini-chromosome maintenance complex-binding protein
	63074081		BS00183592		
TraesCS3A02G097300	63076470			-0.05	Proactivator polypeptide-like 1

TraesCS3A02G097400	63130941			1.51	extracellular glycosidase CRH11-like
TraesCS3A02G097500	63175948			0.00	
TraesCS3A02G097600	63177588			-0.05	probable staphylococcal-like nuclease CAN1 [Aegilops tauschii subsp. tauschii]
TraesCS3A02G097700	63180956			-0.14	alpha-ketoglutarate-dependent dioxygenase alkB homolog 6 [Panicum hallii]
TraesCS3A02G097800	63189474			0.55	probable staphylococcal-like nuclease CAN1 [Aegilops tauschii subsp. tauschii]
TraesCS3A02G097906	63383122			-4.50	pentatricopeptide repeat-containing protein At4g02750
TraesCS3A02G097900	63389133			-6.28	lecithin-cholesterol acyltransferase-like 1
TraesCS3A02G098000	63432665			1.42	SAWADEE HOMEODOMAIN HOMOLOG 2-like
TraesCS3A02G098100	63434267			0.00	lecithin-cholesterol acyltransferase-like 1 [Aegilops tauschii subsp. tauschii]
TraesCS3A02G098200	63472017			-0.07	Protein usf [Hordeum vulgare]
TraesCS3A02G098300	63476129			0.82	
TraesCS3A02G098400	63485329			0.00	Aspartic proteinase nepenthesin-2 [Hordeum vulgare]
TraesCS3A02G098500	63558216			2.85	
TraesCS3A02G098600	63559737			-0.21	ARF guanine-nucleotide exchange factor GNOM-like
TraesCS3A02G098700	63571194			0.77	
TraesCS3A02G098800	63837245			0.90	
TraesCS3A02G098900	63905661			-0.31	Kelch-like protein 5 [Triticum urartu]
TraesCS3A02G099000	64015552			-0.41	
TraesCS3A02G099100	64018774			0.12	Dehydration-responsive element-binding protein 2A [Triticum urartu]
TraesCS3A02G099200	64028173			-0.49	dehydration responsive element binding protein W73 [Triticum aestivum]
TraesCS3A02G099300	64047378			-0.37	protein BREAST CANCER SUSCEPTIBILITY 2 homolog B isoform X4
TraesCS3A02G099400	64278873			2.50	serine/threonine-protein kinase BRI1-like 2
TraesCS3A02G099500	64284579			0.51	dolichol-phosphate mannosyltransferase subunit 3-like
TraesCS3A02G099600	64287121			-0.13	Golgi to ER traffic protein 4 homolog [Aegilops tauschii subsp. tauschii]
TraesCS3A02G099700	64301823			0.90	sigma intracellular receptor 2 [Setaria italica]
TraesCS3A02G099800	64305205			-0.36	serine carboxypeptidase-like 17 [Aegilops tauschii subsp. tauschii]
TraesCS3A02G099900	64315295			-4.27	
TraesCS3A02G100000	64729065			-0.23	putative protein phosphatase 2C 1 [Triticum urartu]
TraesCS3A02G100100	64779052			-0.11	ATP-dependent rRNA helicase spb4
TraesCS3A02G100200	64788196			-0.16	DEAD-box ATP-dependent RNA helicase 40-like isoform
TraesCS3A02G100300	65343379			-0.25	transcription initiation factor TFIID subunit 15b [Hordeum vulgare]

TraesCS3A02G100400	65350151			-0.66	
TraesCS3A02G100500	65360869			0.00	non-classical arabinogalactan protein 30-like
TraesCS3A02G100600	65401536			0.00	non-classical arabinogalactan protein 30-like [Aegilops tauschii subsp. tauschii]
TraesCS3A02G100700	65403689			-0.56	pentatricopeptide repeat-containing protein At5g66520-like [Aegilops tauschii subsp. tauschii]
TraesCS3A02G100800	65433062			1.77	receptor-like protein 12
TraesCS3A02G100900	65466499			0.84	7-deoxyloganetin glucosyltransferase-like [Aegilops tauschii subsp. tauschii]
TraesCS3A02G101000	65477703			0.00	
TraesCS3A02G101100	65701162			0.00	BTR1-A-like protein [Triticum aestivum]
TraesCS3A02G101200	65764573			0.00	BTR1-A-like protein [Triticum aestivum]
TraesCS3A02G101300	65828864			0.00	BTR2-A [Triticum aestivum]
TraesCS3A02G101400	65952061			0.91	E3 ubiquitin-protein ligase [Hordeum vulgare]
TraesCS3A02G101500	65963197			0.00	LRR receptor-like serine/threonine-protein kinase FLS2 [Hordeum vulgare]
TraesCS3A02G101600	65995530			10.00	E3 ubiquitin-protein ligase SINA-like 10
TraesCS3A02G101700	66056962			0.18	L-type lectin-domain containing receptor kinase IV.1-like
TraesCS3A02G101800	66062348			0.05	L-type lectin-domain containing receptor kinase IV.1-like [Aegilops tauschii subsp. tauschii]
TraesCS3A02G101900	66067781			1.57	chlorophyllase-2, chloroplastic-like
TraesCS3A02G102000	66165602			1.46	
TraesCS3A02G102100	66216320			-0.47	
TraesCS3A02G102200	66221705			-0.25	
TraesCS3A02G102300	66227292			8.59	GABA transporter 1-like isoform X2
TraesCS3A02G102400	66249912			0.86	Histone H2A.1 [Triticum urartu]
TraesCS3A02G102500	66254049			1.59	Histone H2B
TraesCS3A02G102600	66271251			0.19	
TraesCS3A02G102700	66297954			0.00	
TraesCS3A02G102800	66353465			0.89	hexose carrier protein HEX6-like
TraesCS3A02G102900	66362604			0.25	
TraesCS3A02G103000	66507059			0.01	
TraesCS3A02G103100	66907810			0.80	
TraesCS3A02G103200	66912987			6.76	F-box protein At5g49610-like
TraesCS3A02G103300	66922161			0.00	

TraesCS3A02G103400	67012730			0.00	
TraesCS3A02G103500	67123770			0.87	protein DELAY OF GERMINATION 1-like
TraesCS3A02G103600	67148999			-0.51	
TraesCS3A02G103700	67151954			-0.30	
TraesCS3A02G103800	67158231			0.14	
TraesCS3A02G103900	67306364			-0.05	
TraesCS3A02G104000	67310163			-0.35	
TraesCS3A02G104100	67359581			1.62	unknown
TraesCS3A02G104300	67383045			0.85	
TraesCS3A02G104400	67785276			2.46	putative transcription factor bHLH041 isoform X1
TraesCS3A02G104500	67910921			0.00	Translation machinery-associated protein 22
TraesCS3A02G104600	68120031			1.50	NDR1/HIN1-like protein 2
TraesCS3A02G104700	68127664			10.50	unknown
TraesCS3A02G104800	68274615			-0.12	proline-rich receptor-like protein kinase PERK9 isoform X3
TraesCS3A02G104900	68283653			-0.29	pentatricopeptide repeat-containing protein At5g16860
TraesCS3A02G105000	68289005			-0.39	Transcription factor IIIB 90 kDa subunit
	68297904		BS00185466		
TraesCS3A02G105100	68867785			1.76	protein indeterminate-domain 7-like
TraesCS3A02G105200	69055191			-6.31	unknown
TraesCS3A02G105300	69098083			14.68	monothiol glutaredoxin-S2-like
TraesCS3A02G105400	69290641			-0.31	Regulatory protein NPR2
TraesCS3A02G105500	69492806			-0.18	protein PHOSPHATE STARVATION RESPONSE 2-like isoform
TraesCS3A02G105600	69504313			0.00	protein IQ-DOMAIN 1-like
TraesCS3A02G105700	69638980			0.83	
TraesCS3A02G105800	69646592			-0.32	
TraesCS3A02G105900	69660730			-0.33	
TraesCS3A02G106000	69949381			0.00	
TraesCS3A02G106100	70476390			9.61	unknown
TraesCS3A02G106200	70702776			-0.32	
TraesCS3A02G106300	70712397			-0.80	
TraesCS3A02G106400	70713666			0.00	
TraesCS3A02G106500	70730050			0.00	

TraesCS3A02G106600	70848330			9.85	1-aminocyclopropane-1-carboxylate synthase 7
TraesCS3A02G106700	71146593			-0.21	
TraesCS3A02G106800	71150103			-5.96	chaperone protein ClpB1-like
TraesCS3A02G106900	71155420			0.00	
TraesCS3A02G107000	71224173			1.01	
TraesCS3A02G107100	71243599			0.00	
TraesCS3A02G107200	71793752			0.48	
TraesCS3A02G107300	71801434			0.00	
TraesCS3A02G107400	72293193			0.25	NAC domain-containing protein 8
TraesCS3A02G107500	72412975			-0.16	
TraesCS3A02G107600	72488099			-0.09	
TraesCS3A02G107700	72492802			-0.47	
TraesCS3A02G107800	72670618			-0.21	unknown
TraesCS3A02G107900	72696221			-0.14	
TraesCS3A02G108000	72896734			1.33	
TraesCS3A02G108100	73512378			1.30	protein IQ-DOMAIN 14-like
TraesCS3A02G108200	73613611			-0.19	
TraesCS3A02G108300	73873079			1.44	momilactone A synthase-like
TraesCS3A02G108400	73929266			0.73	
TraesCS3A02G108500	74236262			-0.36	hexokinase1
TraesCS3A02G108600	74360049			-0.44	
TraesCS3A02G108700	74364854			-0.33	
TraesCS3A02G108800	74427443			0.01	
TraesCS3A02G108900	74781464			-0.41	
TraesCS3A02G109000	75017415			0.80	
TraesCS3A02G109100	75028319			0.01	
TraesCS3A02G109200	75148201			-0.26	
TraesCS3A02G109300	75406115			0.54	
TraesCS3A02G109400	75411043			-0.67	
TraesCS3A02G109500	75545535			8.36	cytokinin oxidase, partia
TraesCS3A02G109600	75565436			0.05	
TraesCS3A02G109700	75590206			-0.32	

TraesCS3A02G109800	75626508			0.00	
TraesCS3A02G109900	75954638			0.06	
TraesCS3A02G110100	77471290			-0.35	protodermal factor 1-like
TraesCS3A02G110200	77474709			0.51	unknown
TraesCS3A02G110300	77483133			-0.04	
TraesCS3A02G110400	77535063			-0.27	
TraesCS3A02G110500	77548371			0.00	
TraesCS3A02G110600	77594911			0.00	
TraesCS3A02G110700	77708465			0.00	
TraesCS3A02G110800	77771743			0.00	
TraesCS3A02G110900	77912201			-0.09	
TraesCS3A02G111000	77939002			-0.23	
TraesCS3A02G111100	78189177			-0.12	
TraesCS3A02G111200	78710172			1.90	WRKY transcription factor
TraesCS3A02G111300	78728574			-0.35	
TraesCS3A02G111400	78899641			0.92	
TraesCS3A02G111500	79184692			-0.28	
TraesCS3A02G111600	79235061			6.41	inactive tetrahydrocannabinolic acid synthase-like
TraesCS3A02G111700	79327173			-0.50	
TraesCS3A02G111800	79334643			0.00	
TraesCS3A02G111900	79379811			0.10	
TraesCS3A02G112000	79396194			3.15	FACT complex subunit SSRP1
TraesCS3A02G112100	79400415			0.06	
TraesCS3A02G112200	79400818			0.19	
TraesCS3A02G112300	79444969			-0.32	homeobox-DDT domain protein RLT3-like isoform X1
TraesCS3A02G112400	79874552			0.00	
TraesCS3A02G112500	80081913			0.00	
TraesCS3A02G112600	80088519			-0.09	ubiquitin-conjugating enzyme E2-like protein
TraesCS3A02G112700	80102807			0.00	
TraesCS3A02G112800	80218476			-0.15	DEAD-box ATP-dependent RNA helicase 39-like
TraesCS3A02G112900	80225715			-1.26	
TraesCS3A02G113000	80288759			0.33	

TraesCS3A02G113100	80412951			-0.21	
TraesCS3A02G113200	80414183			-0.44	
TraesCS3A02G113300	80431989			0.00	
TraesCS3A02G113400	80503366			0.54	
TraesCS3A02G113500	80686016			0.02	
TraesCS3A02G113600	80687298			-0.22	
TraesCS3A02G113700	81086187			0.23	PHD finger protein rhinoceros
TraesCS3A02G113800	81094389			-0.12	
TraesCS3A02G113900	81475905			1.09	probable galactinol--sucrose galactosyltransferase 2
	**81477543**	**HD**	**BS00022624**		
TraesCS3A02G114000	81481700			-0.99	
TraesCS3A02G114100	81662869			0.00	
TraesCS3A02G114200	81692163			-0.30	
TraesCS3A02G114300	81695231			0.19	
TraesCS3A02G114400	81706567			0.03	
TraesCS3A02G114500	82112147			-3.33	unknown
TraesCS3A02G114600	82346228			-1.73	
TraesCS3A02G114700	82392776			-1.41	
TraesCS3A02G114800	82395603			2.11	nuclear transcription factor Y subunit C-6
TraesCS3A02G114900	82439908			1.14	
TraesCS3A02G115000	82743340			0.00	unknown
TraesCS3A02G115100	83021167			-1.59	
TraesCS3A02G115200	83024352			0.00	
TraesCS3A02G115400	83231090			-0.67	
TraesCS3A02G115500	83395843			0.00	
TraesCS3A02G115600	83518688			-0.23	
TraesCS3A02G115700	83690873			0.01	
TraesCS3A02G115800	83720118			-0.22	
TraesCS3A02G115900	83726446			-0.50	
TraesCS3A02G116000	83731800			-0.12	unknown
TraesCS3A02G116100	83774289			0.00	

TraesCS3A02G116200	83977019			0.67	putative WRKY transcription factor 13
TraesCS3A02G116300	84184377			-0.01	TaGI
TraesCS3A02G116400	84649308			0.39	
TraesCS3A02G116500	85270648			0.00	
TraesCS3A02G116600	85371496			0.00	
TraesCS3A02G116700	86121727			-0.17	
TraesCS3A02G116800	86337358			-0.93	
TraesCS3A02G116900	86366135			-0.45	TSL-kinase interacting protein 1-like
TraesCS3A02G117000	86373844			2.26	unknown
TraesCS3A02G117100	86456768			0.15	
TraesCS3A02G117200	86625607			0.00	
TraesCS3A02G117300	87101511			-0.01	
TraesCS3A02G117400	87361021			-0.30	DDT domain-containing protein DDR4-like
TraesCS3A02G117500	87372409			0.90	DNA mismatch repair protein Msh6-2
TraesCS3A02G117600	87775180			0.00	
TraesCS3A02G117700	88189193			-0.08	
TraesCS3A02G117800	88542842			-0.27	
TraesCS3A02G117900	88548543			-0.48	
TraesCS3A02G118000	89572924			0.98	
TraesCS3A02G118100	89666058			0.00	
	90117655		**wmc505**		
TraesCS3A02G118200	90278241			-0.33	
TraesCS3A02G118300	90293197			1.56	
TraesCS3A02G118400	90298975			-0.27	
TraesCS3A02G118500	90364001			0.03	
TraesCS3A02G118600	90790420			-0.22	
TraesCS3A02G118700	90857190			-0.18	
TraesCS3A02G118800	90875218			0.00	
TraesCS3A02G118900	91006697			-0.14	unknown + retrotransposon
TraesCS3A02G119000	91022006			0.14	
TraesCS3A02G119100	91025882			-0.32	

TraesCS3A02G119200	91284700			0.00	
TraesCS3A02G119300	91475181			0.00	
TraesCS3A02G119400	92257934			0.00	
TraesCS3A02G119500	93041129			1.79	
TraesCS3A02G119600	93263631			3.19	acyl transferase 7-like
TraesCS3A02G119700	93263757			2.70	putative PF02458-family acyl transferase 1
TraesCS3A02G119800	93337258			3.71	acyl transferase 7-like
TraesCS3A02G119900	93686897			0.00	
TraesCS3A02G120000	93703505			1.11	unknown
TraesCS3A02G120100	93894059			0.27	
TraesCS3A02G120200	93898636			-0.27	
TraesCS3A02G120300	94115651			1.11	
TraesCS3A02G120400	94182146			0.00	
TraesCS3A02G120500	94186053			0.00	
TraesCS3A02G120600	94253695			0.00	
TraesCS3A02G120700	94857724			0.00	
TraesCS3A02G120800	95016020			0.30	
TraesCS3A02G120900	95017877			-0.16	transposon
TraesCS3A02G121000	95125742			0.00	
TraesCS3A02G121100	95133054			0.00	
TraesCS3A02G121200	95358043			0.00	
TraesCS3A02G121300	95476976			0.04	
TraesCS3A02G121400	95527016			-0.08	
TraesCS3A02G121500	95531831			0.19	Transmembrane protein 19
TraesCS3A02G121600	95535104			0.72	tudor domain-containing protein 3
TraesCS3A02G121700	95539373			-0.44	
TraesCS3A02G121800	95578222			0.00	
TraesCS3A02G121900	95894152			0.00	
TraesCS3A02G122000	95940842			3.23	putative carboxylesterase 13
TraesCS3A02G122100	95944206			-0.15	
TraesCS3A02G122200	96241570			-0.18	

TraesCS3A02G122300	96682449			1.18	Wall-associated receptor kinase-like 20
TraesCS3A02G122400	97046712			0.04	
TraesCS3A02G122500	97467084			-0.49	
TraesCS3A02G122600	97972874			1.81	Gibberellin 3-beta-dioxygenase 2-1
TraesCS3A02G122700	98507031			-1.00	
TraesCS3A02G122800	98513729			-0.15	unknown
TraesCS3A02G122900	98826682			-0.31	Ubiquitin carboxyl-terminal hydrolase
TraesCS3A02G123000	98840535			0.46	
TraesCS3A02G123100	98956012			-0.16	
TraesCS3A02G123200	99268243			0.00	
TraesCS3A02G123300	99296629			0.00	
TraesCS3A02G123400	99387339			0.00	
TraesCS3A02G123500	99587987			0.00	
TraesCS3A02G123600	99622164			0.33	
TraesCS3A02G123700	99994063			1.18	myb family transcription factor EFM
TraesCS3A02G123800	100011353			-0.36	
TraesCS3A02G123900	100014858			2.06	coleoptile phototropism protein 1-like
TraesCS3A02G124100	100188168			-0.24	protein gamma response 1
TraesCS3A02G124200	100345414			-7.18	
TraesCS3A02G124300	100409006			0.20	
TraesCS3A02G124400	100429928			0.27	
TraesCS3A02G124500	100828204			-0.01	
TraesCS3A02G124600	100834044			-0.18	
TraesCS3A02G124700	100841262			0.31	
TraesCS3A02G124800	100842613			-0.04	
TraesCS3A02G124900	100847782			0.00	
TraesCS3A02G125000	100863961			2.33	unknown
TraesCS3A02G125100	100877847			0.61	
TraesCS3A02G125200	100880788			-0.12	
TraesCS3A02G125300	100885506			0.01	chloride channel protein CLC-c-like
TraesCS3A02G125400	100946636			0.00	

TraesCS3A02G125500	100958315			1.39	
TraesCS3A02G125600	100975593			0.00	
TraesCS3A02G125700	101099681			9.42	zeatin O-glucosyltransferase 3
TraesCS3A02G125800	101567459			3.34	UDP-glycosyltransferase 73C5-like
TraesCS3A02G125900	101608015			1.50	
TraesCS3A02G126000	101776593			3.63	unknown
TraesCS3A02G126100	101866028			0.00	
TraesCS3A02G126200	101891087			0.00	
TraesCS3A02G126300	101893744			0.24	
TraesCS3A02G126600	102115581			-6.49	
TraesCS3A02G126800	102274744			-9.54	
TraesCS3A02G126900	102359953			0.00	
TraesCS3A02G127000	102435711			6.99	unknown
TraesCS3A02G127100	102554499			0.65	
TraesCS3A02G127200	102615927			-0.17	
TraesCS3A02G127300	102623417			10.89	uncharacterized acetyltransferase At3g50280-like
TraesCS3A02G127400	102677471			-0.25	
TraesCS3A02G127500	102686298			0.00	
TraesCS3A02G127600	102794069			0.00	
TraesCS3A02G127700	103618043			0.11	
TraesCS3A02G127800	104414814			-0.25	
TraesCS3A02G127900	104419888			-0.25	
TraesCS3A02G128000	104557653			-0.25	
TraesCS3A02G128100	105026576			0.00	
TraesCS3A02G128200	105111804			1.88	protein kinase PINOID 2
TraesCS3A02G128300	105712838			0.00	
TraesCS3A02G128400	105944440			0.00	
TraesCS3A02G128500	105946479			-0.46	
TraesCS3A02G128600	106303465			-0.37	
TraesCS3A02G128700	106396973			0.68	BTB/POZ and MATH domain-containing protein 1-like
TraesCS3A02G128800	106401457			-0.29	

TraesCS3A02G128900	106433266			0.27	
TraesCS3A02G129000	106434735			-0.40	ABC transporter C family member 3-like
	106434896		BS00022148		
TraesCS3A02G129100	106555889			0.47	Glyoxalase
TraesCS3A02G129200	106657461			-0.36	
TraesCS3A02G129300	106659927			0.10	
TraesCS3A02G129400	106660848			0.00	
TraesCS3A02G129500	106676627			0.00	
TraesCS3A02G129600	106834395			1.23	
TraesCS3A02G129700	107043940			3.80	unknown
TraesCS3A02G129800	107047681			0.74	
TraesCS3A02G129900	107052572			0.28	
TraesCS3A02G130000	107076189			-0.07	
TraesCS3A02G130100	107316698			-0.24	
TraesCS3A02G130200	107397424			-0.13	
TraesCS3A02G130300	107402918			-0.14	phosphoenolpyruvate/phosphate translocator 3, chloroplastic-like
TraesCS3A02G130400	107416364			0.00	
TraesCS3A02G130500	107418238			-0.12	
TraesCS3A02G130600	107528741			0.00	
TraesCS3A02G130700	107565870			0.00	
TraesCS3A02G130800	107619668			2.12	unknown
TraesCS3A02G130900	107653078			-0.57	unknown
TraesCS3A02G131000	107732788			-0.32	
TraesCS3A02G131100	107744078			0.00	
TraesCS3A02G131200	107755822			1.34	probable carboxylesterase 15
TraesCS3A02G131300	107758099			0.00	
TraesCS3A02G131400	107863262			6.84	RING-H2 finger protein ATL65-like
TraesCS3A02G131500	107975081			2.25	RING-H2 finger protein ATL39-like
TraesCS3A02G131600	108033015			2.09	jacalin-related lectin 19-like
TraesCS3A02G131700	108373401			0.29	protein SPEAR2
TraesCS3A02G131800	108457488			0.14	

TraesCS3A02G131900	108729433			0.72	basic leucine zipper 2-like
TraesCS3A02G132000	108744935			2.08	
TraesCS3A02G132100	108879584			-0.44	
TraesCS3A02G132200	108918715			-1.83	unknown
TraesCS3A02G132300	108952141			0.56	
TraesCS3A02G132400	109149213			-0.14	
TraesCS3A02G132500	109297969			0.52	
TraesCS3A02G132600	109341955			1.20	
TraesCS3A02G132700	109867886			0.96	
TraesCS3A02G132800	109869449			0.00	
TraesCS3A02G132900	109871699			0.04	
TraesCS3A02G133000	110000112			1.60	putative cyclin-dependent kinase F-2
TraesCS3A02G133100	110069451			1.39	
TraesCS3A02G133200	110151139			0.00	
TraesCS3A02G133300	110309461			-0.27	
TraesCS3A02G133400	110332577			0.88	gibberellin 2-beta-dioxygenase 1-like
TraesCS3A02G133500	110680985			-0.29	unknown
TraesCS3A02G133600	110777434			-0.09	
TraesCS3A02G133700	110788290			-0.03	
TraesCS3A02G133900	111103486			1.91	
TraesCS3A02G134000	111105593			0.94	
TraesCS3A02G134100	111243704			-0.24	
TraesCS3A02G134200	111550279			0.82	
TraesCS3A02G134300	111555102			0.03	
TraesCS3A02G134400	111807173			0.00	
TraesCS3A02G134500	111824319			0.71	
TraesCS3A02G134600	111961695			1.39	
TraesCS3A02G134700	112296815			0.00	
TraesCS3A02G134800	112409703			0.00	
TraesCS3A02G134900	112917876			-0.36	peptide-N4-(N-acetyl-beta-glucosaminyl)asparagine amidase A-like
TraesCS3A02G135000	112925350			-1.73	

TraesCS3A02G135100	112936618			-0.12	
TraesCS3A02G135200	112943978			0.00	
TraesCS3A02G135300	112947180			0.11	
TraesCS3A02G135400	112950898			0.15	
TraesCS3A02G135500	112957446			-0.29	
TraesCS3A02G135600	112961349			1.02	
TraesCS3A02G135700	113340512			1.39	putative CBL-interacting protein kinase 13
TraesCS3A02G135800	113354731			-0.08	
TraesCS3A02G135900	113365783			0.00	
TraesCS3A02G136000	113709059			0.00	
TraesCS3A02G136100	113709769			0.48	
TraesCS3A02G136200	113870607			0.00	
TraesCS3A02G136300	113921279			1.74	
TraesCS3A02G136500	114041077			-0.19	SHAGGY-like kinase (related to AtBIN2)
TraesCS3A02G136600	114047082			0.56	
TraesCS3A02G136700	114237731			1.25	enoyl-CoA delta isomerase 2, peroxisomal-like
TraesCS3A02G136800	114239777			-0.32	
TraesCS3A02G136900	114421566			0.09	
TraesCS3A02G137000	114422943			-0.32	
TraesCS3A02G137100	114426404			-0.25	
TraesCS3A02G137200	114433342			0.00	
TraesCS3A02G137300	114443998			0.00	
TraesCS3A02G137400	114540087			0.00	
TraesCS3A02G137500	114567344			-0.26	
TraesCS3A02G137600	114568067			0.62	UMP-CMP kinase 1-like
TraesCS3A02G137700	114695981			0.00	
TraesCS3A02G137900	114712488			0.83	
TraesCS3A02G138000	114770298			2.52	protein GRAVITROPIC IN THE LIGHT 1
TraesCS3A02G138100	114793466			-0.49	
TraesCS3A02G138200	115162195			0.32	Subtilisin-like protease
TraesCS3A02G138300	115389817			-2.05	Subtilisin-like protease

TraesCS3A02G138400	115839464			-1.83	
TraesCS3A02G138500	115847640			0.00	
TraesCS3A02G138600	116074087			-2.07	
TraesCS3A02G138700	116100432			7.31	unknown
TraesCS3A02G138900	116284377			0.08	
TraesCS3A02G139000	116518694			0.07	
TraesCS3A02G139100	116525681			0.00	
TraesCS3A02G139200	116527572			-0.16	serine/threonine-protein phosphatase 5
TraesCS3A02G139300	117227850			0.57	
TraesCS3A02G139400	117788639			0.00	
TraesCS3A02G139500	117805633			-0.09	
TraesCS3A02G139600	117827720			-0.05	
TraesCS3A02G139700	118546170			4.39	RING-H2 finger protein ATL39
TraesCS3A02G139800	118546567			0.00	
TraesCS3A02G139900	118566831			1.35	
TraesCS3A02G140000	118587495			2.17	RING-H2 finger protein ATL74-like
TraesCS3A02G140100	119137149			0.71	
TraesCS3A02G140200	119470233			-0.27	
TraesCS3A02G140300	119473835			-0.14	uncharacterized aarF domain-containing protein kinase 1 isoform X3
TraesCS3A02G140400	119485894			0.00	
TraesCS3A02G140500	119686627			0.20	
TraesCS3A02G140600	119693993			-0.30	
TraesCS3A02G140700	119697917			0.90	
TraesCS3A02G140800	120175318			0.00	
TraesCS3A02G140900	120223456			0.68	
TraesCS3A02G141000	120227737			0.00	
TraesCS3A02G141100	120266288			0.85	GDSL esterase/lipase
TraesCS3A02G141200	120296042			-0.63	
TraesCS3A02G141300	120357124			0.74	
TraesCS3A02G141400	120692119			8.13	putative esterase
TraesCS3A02G141500	120801205			0.79	

TraesCS3A02G141600	121235606			0.00	
TraesCS3A02G141700	121317335			0.00	
TraesCS3A02G141800	121327477			0.00	
TraesCS3A02G141900	121387722			-0.39	Putative SWI/SNF-related matrix-associated actin-dependent regulator of chromatin subfamily A member 3-like 2
TraesCS3A02G142000	121479361			0.00	
TraesCS3A02G142100	121484171			-0.43	
TraesCS3A02G142200	121644384			-0.02	
TraesCS3A02G142300	122208067			-1.01	
TraesCS3A02G142400	122222409			0.00	
TraesCS3A02G142500	122229366			-0.69	
TraesCS3A02G142600	122464482			7.84	LINE-1 retrotransposable element ORF2 protein
TraesCS3A02G142700	123045617			0.60	
TraesCS3A02G142800	123277990			-0.25	
TraesCS3A02G142900	123284926			0.79	transcription factor bHLH30-like
TraesCS3A02G143000	123326283			-0.24	
TraesCS3A02G143100	124172881			1.87	FT-like protein (FT2)
TraesCS3A02G143200	124179766			-0.21	
TraesCS3A02G143300	124187662			-0.33	
TraesCS3A02G143400	124655539			1.52	
TraesCS3A02G143500	124658180			-0.20	
TraesCS3A02G143600	125092210			-0.32	
TraesCS3A02G143700	125368934			0.00	
TraesCS3A02G143800	125422646			1.07	
TraesCS3A02G143900	125484253			-0.01	
TraesCS3A02G144000	125488067			0.28	probable F-box protein At3g61730
TraesCS3A02G144100	125496055			-0.24	
TraesCS3A02G144200	126035274			0.00	
TraesCS3A02G144400	126119669			0.00	
TraesCS3A02G144500	127071707			0.00	
TraesCS3A02G144600	127088113			0.00	

TraesCS3A02G144700	127192010			-0.33	
TraesCS3A02G144800	127271681			0.13	
TraesCS3A02G144900	127353948			-0.69	
TraesCS3A02G145000	127820224			0.00	
TraesCS3A02G145100	127856285			-6.16	
TraesCS3A02G145200	127865530			7.56	unknown
TraesCS3A02G145300	127870047			1.23	
TraesCS3A02G145400	127959332			0.00	
TraesCS3A02G145500	127961941			-7.71	
TraesCS3A02G145600	127965591			0.00	
TraesCS3A02G145700	127973254			-0.13	
TraesCS3A02G145900	128082220			-0.12	
TraesCS3A02G146000	128138163			0.00	
TraesCS3A02G146100	128150132			-0.38	putative glycerol-3-phosphate acyltransferase 3
TraesCS3A02G146200	128152904			0.05	
TraesCS3A02G146300	128164849			0.63	
TraesCS3A02G146400	128216943			0.02	
TraesCS3A02G146500	128287161			0.57	
TraesCS3A02G146600	128293099			-0.13	
TraesCS3A02G146700	128302328			-0.34	
TraesCS3A02G146800	128522801			0.47	
TraesCS3A02G146900	128539724			0.03	
TraesCS3A02G147000	128559103			0.24	
TraesCS3A02G147100	128564553			-0.53	
TraesCS3A02G147200	128578407			1.75	glycerol-3-phosphate acyltransferase 1
TraesCS3A02G147300	128582053			-0.20	
TraesCS3A02G147400	128658771			0.00	
TraesCS3A02G147500	128716737			0.05	
TraesCS3A02G147600	130834630			0.86	
TraesCS3A02G147700	130836644			-0.16	
TraesCS3A02G147800	130842186			-1.17	

TraesCS3A02G147900	130851402			-0.06	
TraesCS3A02G148000	130896344			2.03	
TraesCS3A02G148100	130966615			1.26	putative E3 ubiquitin-protein ligase LIN-1 isoform X1
TraesCS3A02G148200	131343237			-0.03	
TraesCS3A02G148300	131348327			-0.06	
TraesCS3A02G148400	131402757			0.14	
TraesCS3A02G148500	131415189			0.06	
TraesCS3A02G148600	131422559			0.11	
TraesCS3A02G148700	131458286			-0.08	
TraesCS3A02G148800	131646148			0.39	
TraesCS3A02G148900	131749774			-0.07	
TraesCS3A02G149000	132581440			0.99	ethylene-responsive transcription factor CRF1-like
TraesCS3A02G149100	134234662			-0.16	
TraesCS3A02G149200	134237719			1.28	
TraesCS3A02G149300	134240740			-0.11	mitochondrial carrier protein MTM1-like
TraesCS3A02G149400	134245584			0.02	
TraesCS3A02G149500	134600543			0.00	
TraesCS3A02G149600	134613198			0.00	
TraesCS3A02G149700	135378286			0.00	
TraesCS3A02G149800	135968322			-0.32	
TraesCS3A02G149900	136427933			1.02	mitochondrial arginine transporter BAC2
TraesCS3A02G150000	136526562			0.51	no hits
TraesCS3A02G150100	136528443			-0.20	unknown
TraesCS3A02G150200	136567573			-0.05	
TraesCS3A02G150300	137574043			-0.15	
TraesCS3A02G150400	137591590			-0.12	GTP binding
TraesCS3A02G150500	137600605			-9.23	
TraesCS3A02G150600	137609236			-0.59	
TraesCS3A02G150700	137785828			0.00	
TraesCS3A02G150800	137787132			-0.07	
TraesCS3A02G150900	137912166			-0.21	

TraesCS3A02G151000	138284334			1.44	probable membrane-associated kinase regulator 2
TraesCS3A02G151100	140003720			-0.28	peptidyl-prolyl cis-trans isomerase, chloroplastic
TraesCS3A02G151200	140040910			-0.33	
TraesCS3A02G151300	140050261			-8.18	
TraesCS3A02G151400	140805091			0.00	
TraesCS3A02G151500	140956483			0.00	
TraesCS3A02G151600	141008416			-0.12	
TraesCS3A02G151700	141041357			-0.18	U11/U12 small nuclear ribonucleoprotein 59 kDa protein isoform X2
TraesCS3A02G151800	141910098			1.57	transcription repressor OFP3-like
TraesCS3A02G151900	142573563			0.04	
TraesCS3A02G152000	142579172			-0.01	
TraesCS3A02G152100	142878674			1.33	
TraesCS3A02G152200	142890247			0.06	PERK1
TraesCS3A02G152300	142896448			-0.36	
TraesCS3A02G152500	142923164			0.98	
TraesCS3A02G152600	142976623			0.00	
TraesCS3A02G152700	143017324			1.25	(+)-menthofuran synthase-like
TraesCS3A02G152800	143470691			0.00	
TraesCS3A02G153000	143533072			0.86	
TraesCS3A02G153100	143541890			-0.01	
TraesCS3A02G153200	144009541			0.00	
TraesCS3A02G153300	144149003			1.17	
TraesCS3A02G153400	144152212			1.30	Glyoxylate reductase
TraesCS3A02G153500	144154998			1.05	
TraesCS3A02G153600	144164850			-0.07	
TraesCS3A02G153700	144433736			-0.34	Myb-related protein 3R-1
TraesCS3A02G153800	144448392			0.00	
TraesCS3A02G153900	144542938			-0.19	
TraesCS3A02G154000	145250370			-0.15	
TraesCS3A02G154100	145557726			0.00	
TraesCS3A02G154200	145620582			-0.08	unknown

TraesCS3A02G154300	145634971			0.00	
TraesCS3A02G154400	145667222			0.74	abscisic acid receptor PYL2
TraesCS3A02G154500	146386030			0.07	
TraesCS3A02G154600	146394197			-0.35	
TraesCS3A02G154700	146559102			-0.31	
TraesCS3A02G154800	146562236			1.92	thioesterase family protein
TraesCS3A02G154900	146601447			1.89	putative E3 ubiquitin-protein ligase LIN
TraesCS3A02G155000	146772726			3.75	putative unclassified retrotransposon protein
TraesCS3A02G155100	147283673			-0.10	
TraesCS3A02G155200	147789276			-0.16	IAA3
TraesCS3A02G155300	147812738			0.40	
TraesCS3A02G155400	148603253			-0.31	
TraesCS3A02G155500	148615573			8.71	VAN3-binding protein-like
TraesCS3A02G155600	148617573			-0.16	
TraesCS3A02G155700	149209877			0.00	
TraesCS3A02G155800	149564155			1.52	probable aquaporin TIP4-2
TraesCS3A02G155900	149715311			0.00	
TraesCS3A02G156000	149985637			1.42	protein JINGUBANG-like
TraesCS3A02G156100	150711373			-0.21	
TraesCS3A02G156200	150718366			-0.13	
TraesCS3A02G156300	151314356			-0.16	
TraesCS3A02G156400	151322267			1.21	unknown
TraesCS3A02G156500	151642540			-0.33	ABI8
TraesCS3A02G156600	151675660			-0.18	unknown
TraesCS3A02G156700	152000555			0.00	
TraesCS3A02G156800	153076188			0.11	
TraesCS3A02G156900	153362219			-0.45	
TraesCS3A02G157000	153594473			-0.36	
TraesCS3A02G157100	154029658			-8.03	serine/threonine-protein kinase RHS3-like (root hairs)
TraesCS3A02G157200	154032915			0.18	
TraesCS3A02G157300	154072477			-0.59	

TraesCS3A02G157400	154383740			0.00	
TraesCS3A02G157500	154658571			-0.67	
TraesCS3A02G157600	154741112			-0.99	unknown
TraesCS3A02G157700	155057280			0.00	
TraesCS3A02G157800	155057280			-0.15	
TraesCS3A02G157900	155420436			0.00	reticulon-like protein B21
TraesCS3A02G158000	155429154			-0.12	
TraesCS3A02G158100	155825149			-0.07	
TraesCS3A02G158200	155826735			-0.29	
TraesCS3A02G158300	156124586			-0.06	
TraesCS3A02G158400	156230024			-0.44	WPP domain-interacting tail-anchored protein 1
TraesCS3A02G158500	156237883			-0.48	
TraesCS3A02G158600	156267711			-0.10	
TraesCS3A02G158700	157021716			-0.19	
TraesCS3A02G158800	157031841			0.89	unknown
TraesCS3A02G158900	157043589			0.52	
TraesCS3A02G159000	157347435			0.00	
TraesCS3A02G159100	158170503			-0.04	
TraesCS3A02G159200	158467595			-0.40	ARF1
TraesCS3A02G159400	159345301			-0.26	
TraesCS3A02G159500	159347039			-0.22	
TraesCS3A02G159600	159422674			-0.24	
TraesCS3A02G159700	159431340			0.91	
TraesCS3A02G159900	159835197			-0.01	
TraesCS3A02G160000	159995394			0.00	
TraesCS3A02G160100	160016413			0.00	
TraesCS3A02G160200	160074349			0.33	COP9 signalosome complex subunit 6a [Triticum urartu]
TraesCS3A02G160300	160076442			-0.18	
TraesCS3A02G160400	160295461			-8.72	isoflavone reductase homolog
TraesCS3A02G160500	160506568			1.56	unknown
TraesCS3A02G160600	161443608			-0.02	

TraesCS3A02G160700	161446234			-0.89	
TraesCS3A02G160800	161449857			13.54	protein NUCLEAR FUSION DEFECTIVE 6, chloroplastic/mitochondrial-like isoform X1
TraesCS3A02G160900	161451259			-0.34	DDB1- and CUL4-associated factor 13 [Aegilops tauschii subsp. tauschii]
TraesCS3A02G161000	161460235			0.49	
TraesCS3A02G161100	162752691			0.00	
TraesCS3A02G161200	162756053			-0.11	
TraesCS3A02G161300	162761485			-0.33	triose phosphate translocator [Triticum aestivum]
TraesCS3A02G161400	163621322			0.10	
TraesCS3A02G161500	163693202			0.00	
TraesCS3A02G161600	163792995			0.05	
TraesCS3A02G161800	163929413			-0.15	
TraesCS3A02G161900	164369931			-0.30	
TraesCS3A02G162000	164403707			-0.15	
TraesCS3A02G162100	164419424			0.00	
TraesCS3A02G162200	164610874			-6.97	unknown
TraesCS3A02G162300	164614843			0.00	
TraesCS3A02G162400	164718651			0.00	
TraesCS3A02G162500	164802465			0.00	
TraesCS3A02G162600	164863015			0.00	
TraesCS3A02G162700	164910707			0.00	
TraesCS3A02G162800	164928880			-1.49	unknown
TraesCS3A02G162900	165087589			0.00	
TraesCS3A02G163000	165320368			1.82	unknown
TraesCS3A02G163100	165328563			-0.24	
TraesCS3A02G163200	165333707			-0.05	tRNA(His) guanylyltransferase 2-like
TraesCS3A02G163300	165415769			-0.08	
TraesCS3A02G163400	165843496			8.94	unknown
TraesCS3A02G163500	166149246			0.00	
TraesCS3A02G163700	166925104			-0.31	
TraesCS3A02G163900	168590929			0.12	
TraesCS3A02G164100	168832726			-0.46	

TraesCS3A02G164000	168833301			-0.02	
TraesCS3A02G164200	168837089		BS00021976	-0.21	shaggy-related protein kinase alpha [Brachypodium distachyon] = BS00021976
TraesCS3A02G164300	169226560			2.49	MFS18 protein-like [Aegilops tauschii subsp. tauschii]
TraesCS3A02G164400	169290148			0.00	
TraesCS3A02G164500	169839469			5.86	unknown
TraesCS3A02G164600	169845024			-0.34	
TraesCS3A02G164700	169852539			-0.08	
TraesCS3A02G164800	170226838			1.11	unknown
TraesCS3A02G164900	170233390			-0.42	
TraesCS3A02G165000	170244696			-0.17	
TraesCS3A02G165100	170383582			-0.27	
TraesCS3A02G165200	170388365			-0.27	
TraesCS3A02G165300	170390397			0.00	
TraesCS3A02G165400	170485669			0.79	heavy metal-associated isoprenylated plant protein 43-like
TraesCS3A02G165500	170648445			0.55	
TraesCS3A02G165600	170658582			0.33	
TraesCS3A02G165700	170741455			0.53	
TraesCS3A02G165800	170783633			0.01	unknown
TraesCS3A02G165900	170811477			1.89	expansin EXPA5
TraesCS3A02G166002	170978175			-0.02	
TraesCS3A02G166000	170992733			0.91	
TraesCS3A02G166100	171033857			0.63	
TraesCS3A02G166200	171127374			0.67	
TraesCS3A02G166300	171137518			0.28	
TraesCS3A02G166400	171148800			-1.15	signal recognition particle 14 kDa protein
TraesCS3A02G166500	171173173			0.39	
TraesCS3A02G166600	171174416			-0.14	isocitrate dehydrogenase [NADP] isoform X2
TraesCS3A02G166700	171209686			0.00	
TraesCS3A02G166900	171255579			0.89	
TraesCS3A02G167000	171548939			6.94	S-type anion channel SLAH1-like
TraesCS3A02G167100	172629517			-0.20	putative brassinosteroid receptor [Oryza sativa Japonica Group]

TraesCS3A02G167200	172963326			1.00	WRKY transcription factor 6
TraesCS3A02G167300	173942316			0.00	
TraesCS3A02G167400	173943797			-0.21	
TraesCS3A02G167700	174283442			0.04	unknown
TraesCS3A02G167800	174292756			1.10	unknown
TraesCS3A02G167900	174542475			-0.67	unknown
TraesCS3A02G168000	174768031			0.00	
TraesCS3A02G168100	175237609			-0.51	
TraesCS3A02G168200	175632606			-0.39	
TraesCS3A02G168300	175808526			0.90	unknown
TraesCS3A02G168400	175844695			0.00	
TraesCS3A02G168500	175859332			0.33	
TraesCS3A02G168600	175920771			-0.65	
TraesCS3A02G168800	176143399			-0.29	
TraesCS3A02G168900	176932023			-0.14	
TraesCS3A02G169000	176990481			-7.97	unknown
TraesCS3A02G169100	177086937			0.07	
TraesCS3A02G169200	177104743			-0.24	
TraesCS3A02G169600	177702531			0.16	probable L-type lectin-domain containing receptor kinase S.5
TraesCS3A02G169800	177894086			-0.88	
TraesCS3A02G169900	178118523			-0.09	
TraesCS3A02G170000	178130748			0.06	
TraesCS3A02G170100	178133754			-0.07	
TraesCS3A02G170200	178147889			0.00	
TraesCS3A02G170300	178245198			-0.14	
TraesCS3A02G170400	178251802			-0.11	
TraesCS3A02G170500	178254443			0.00	
TraesCS3A02G170600	178256071			0.01	
TraesCS3A02G170700	178985803			-7.74	zinc finger protein NUTCRACKER-like
TraesCS3A02G170800	179534751			0.08	
TraesCS3A02G170900	180743665			0.00	

TraesCS3A02G171000	181087402			0.00	
TraesCS3A02G171100	184214128			0.00	
TraesCS3A02G171200	185166522			0.00	
TraesCS3A02G171300	185173843			0.79	
TraesCS3A02G171400	185177131			-0.34	
TraesCS3A02G171500	186040029			6.31	ANXUR1 male factors controlling pollen tube behavior
TraesCS3A02G171600	186043009			-0.27	
TraesCS3A02G171800	186707977			0.00	
TraesCS3A02G172000	186745878			-0.15	
TraesCS3A02G172100	186750246			1.77	unknown
TraesCS3A02G172200	186845109			0.74	
TraesCS3A02G172300	187247733			1.33	trinucleotide repeat-containing gene 18 protein-like
TraesCS3A02G172400	187250391			-0.51	
TraesCS3A02G172500	187255027			0.00	
TraesCS3A02G172600	187305871			6.91	unknown
TraesCS3A02G172700	187432157			0.00	
TraesCS3A02G172800	188101307			0.00	
TraesCS3A02G172900	189275871			-0.35	GTP-binding protein SAR1A-like
TraesCS3A02G173000	189316375			0.11	
TraesCS3A02G173100	191006045			-8.02	
TraesCS3A02G173200	191252409			-0.34	unknown
TraesCS3A02G173300	191527521			-0.19	Topless
TraesCS3A02G173400	191744853			0.00	
TraesCS3A02G173500	191837340			-0.77	
TraesCS3A02G173600	191840265			0.41	
TraesCS3A02G173700	191856550			-0.38	
TraesCS3A02G173800	191857329			0.00	
TraesCS3A02G174000	191889546			0.00	
TraesCS3A02G174100	192816217			1.60	pectinesterase-like
TraesCS3A02G174200	192849290			0.03	
TraesCS3A02G174300	192853613			-1.88	

TraesCS3A02G174400	192854503			0.13	
TraesCS3A02G174500	193597951			-0.17	
TraesCS3A02G174600	193873752			-0.22	
TraesCS3A02G174700	193915579			0.00	
TraesCS3A02G174800	194061628			-0.10	
TraesCS3A02G174900	194705893			-0.38	RNA-binding protein 25-like
TraesCS3A02G175000	194804163			-0.22	
TraesCS3A02G175100	195155807			0.00	
TraesCS3A02G175200	195171635			0.15	
TraesCS3A02G175300	195184518			-0.82	dynein light chain 1, cytoplasmic
TraesCS3A02G175400	195188996			0.12	
TraesCS3A02G175500	195453879			-0.25	
TraesCS3A02G175600	195456777			-0.18	
TraesCS3A02G175700	195462866			-0.04	zinc finger CCCH domain-containing protein 4
TraesCS3A02G175800	196180861			-0.40	
TraesCS3A02G175900	196195769			-0.25	
TraesCS3A02G176000	196419554			0.66	
TraesCS3A02G176100	196761940			2.24	flowering-promoting factor 1-like protein 1
TraesCS3A02G176200	197092140			0.00	
TraesCS3A02G176300	197428753			-0.04	
TraesCS3A02G176400	197433113			-0.35	
TraesCS3A02G176500	197849345			-0.28	
TraesCS3A02G176600	197860295			-0.04	
TraesCS3A02G176700	198034565			-0.16	
TraesCS3A02G176800	198181297			-1.88	
TraesCS3A02G176900	198489602			-0.18	
TraesCS3A02G177000	198836313			0.10	
TraesCS3A02G177100	199145442			-0.30	pentatricopeptide repeat-containing protein At3g06920
TraesCS3A02G177200	199412423			0.00	
TraesCS3A02G177300	199688875			-0.55	CASPARIAN STRIP INTEGRITY FACTOR 1
TraesCS3A02G177400	199694319			-0.26	

TraesCS3A02G177500	200810372			-0.25	
TraesCS3A02G177600	201475006			1.52	unknown
TraesCS3A02G177700	201498893			-0.43	
TraesCS3A02G177800	201506461			-0.36	
TraesCS3A02G177900	202584455			0.00	
TraesCS3A02G178000	202588435			-0.15	
TraesCS3A02G178100	202634326			-0.20	
TraesCS3A02G178200	202638266			-0.29	
TraesCS3A02G178300	202826530			7.96	putative cyclin-dependent kinase F-2
TraesCS3A02G178400	202827156			0.33	
TraesCS3A02G178500	202984528			0.00	
TraesCS3A02G178600	203654393			-0.35	protein HOTHEAD-like
TraesCS3A02G178700	203983146			-0.10	
TraesCS3A02G178800	204821537			0.26	
TraesCS3A02G178900	204865876			-0.37	
TraesCS3A02G179000	204882919			-0.05	
TraesCS3A02G179100	205689861			-0.10	
TraesCS3A02G179200	206623114			-0.09	
TraesCS3A02G179300	207241844			0.00	
TraesCS3A02G179400	207829763			0.25	
TraesCS3A02G179500	207842123			0.43	
TraesCS3A02G179600	207845330			-0.25	
TraesCS3A02G179700	208031588			-0.15	
TraesCS3A02G179800	208066482			-0.56	
TraesCS3A02G179900	208080790			0.09	
TraesCS3A02G180000	208085351			1.45	
TraesCS3A02G180100	208088981			0.00	
TraesCS3A02G180200	208221170			1.06	
TraesCS3A02G180300	208234139			0.37	
TraesCS3A02G180400	208237786			-0.64	
TraesCS3A02G180500	208241293			-0.35	

TraesCS3A02G180600	208246099			-0.11	cyclic dof factor (CDF) 2-like
TraesCS3A02G180700	208454984			2.27	unknown
TraesCS3A02G180800	208637892			-7.82	
TraesCS3A02G180900	208724568			-0.07	
TraesCS3A02G181000	208735649			2.54	PHYTOCHROME KINASE SUBSTRATE 4
TraesCS3A02G181100	208801195			-0.13	
TraesCS3A02G181200	209402146			-0.11	
TraesCS3A02G181300	209525298			-2.58	
TraesCS3A02G181400	209602410			2.09	geranylgeranyl diphosphate reductase, chloroplastic-like
TraesCS3A02G181500	209653024			-0.22	
TraesCS3A02G181600	210092865			-0.08	
TraesCS3A02G181700	210125457			0.00	
TraesCS3A02G181800	210246833			-0.06	
TraesCS3A02G181900	210258397			-0.09	
TraesCS3A02G182000	210932388			-0.17	
TraesCS3A02G182100	211188157			1.16	E3 ubiquitin-protein ligase EL5-like
TraesCS3A02G182200	211503919			-0.21	
TraesCS3A02G182300	211952697			0.00	
TraesCS3A02G182400	211953911			0.68	unknown
TraesCS3A02G182500	211955321			0.01	
TraesCS3A02G182600	212086482			0.57	
TraesCS3A02G182700	212087787			0.06	
TraesCS3A02G182800	212109352			-0.19	
TraesCS3A02G182900	212112431			-0.12	
TraesCS3A02G183100	212531004			-0.05	
TraesCS3A02G183200	212702120			-0.03	
TraesCS3A02G183300	212706091			0.00	
TraesCS3A02G183400	212712042			0.00	
TraesCS3A02G183500	212740793			-0.05	
TraesCS3A02G183600	212768626			-0.35	
TraesCS3A02G183700	213129151			0.01	

TraesCS3A02G183800	213256188			0.57	
TraesCS3A02G183900	213735741			0.43	
TraesCS3A02G184000	214116286			-0.15	
TraesCS3A02G184200	214287963			-0.17	
TraesCS3A02G184300	215202922			0.17	
TraesCS3A02G184400	215471906			4.04	serine/threonine-protein kinase-like protein At5g23170
TraesCS3A02G184500	215478994			-0.24	
TraesCS3A02G184700	215485340			-1.08	probable peptide/nitrate transporter At3g43790 isoform
TraesCS3A02G184800	215950065			-0.42	
TraesCS3A02G184900	216133520			1.12	Alpha-glucosidase yihQ
TraesCS3A02G185000	216465668			3.14	protein MIZU-KUSSEI 1-like
TraesCS3A02G185200	216937529			0.00	
TraesCS3A02G185300	217713628			0.00	
TraesCS3A02G185400	217817377			-0.21	
TraesCS3A02G185500	217820700			-0.08	
TraesCS3A02G185600	217958846			0.00	
TraesCS3A02G185700	217981422			-0.01	
TraesCS3A02G185800	218117459			0.10	
TraesCS3A02G185900	218728050			0.70	
TraesCS3A02G186000	219725917			0.00	
TraesCS3A02G186100	219793803			0.06	
TraesCS3A02G186200	219797550			-0.22	
TraesCS3A02G186300	220016030			0.00	
TraesCS3A02G186400	220886752			-0.22	
TraesCS3A02G186500	221277371			-0.20	
TraesCS3A02G186600	221667628			0.04	
TraesCS3A02G186700	221923658			0.24	
TraesCS3A02G186800	221927970			1.21	unknown
TraesCS3A02G186900	221930752			8.16	Early nodulin-like protein 1
TraesCS3A02G187000	221946541			0.22	
TraesCS3A02G187100	222042031			-0.28	

TraesCS3A02G187200	222452133			-0.23	
TraesCS3A02G187300	223521136			-0.44	
TraesCS3A02G187400	223705484			0.98	
TraesCS3A02G187500	224363400			-0.20	
TraesCS3A02G187600	224377624			0.11	
TraesCS3A02G187700	224969259			7.42	unknown
TraesCS3A02G187800	225893370			2.21	transcriptional activator Myb-like
TraesCS3A02G187900	227364659			-0.17	
TraesCS3A02G188000	227407930			-0.04	
TraesCS3A02G188100	227632777			4.86	apocytochrome b
TraesCS3A02G188200	227637631			0.00	
TraesCS3A02G188300	227645744			0.00	
TraesCS3A02G188400	227908994			1.03	
TraesCS3A02G188500	227921423			-0.38	
TraesCS3A02G188600	229177381			0.00	
TraesCS3A02G188700	230091436			-0.67	
TraesCS3A02G188800	230227535			0.35	
TraesCS3A02G188900	230999664			-0.29	Crossover junction endonuclease MUS81
TraesCS3A02G189000	231014310			-0.21	
TraesCS3A02G189100	231039843			0.41	
TraesCS3A02G189200	231669384			0.50	
TraesCS3A02G189300	231671439			-0.08	
TraesCS3A02G189400	232268975			-0.15	
TraesCS3A02G189500	233021585			0.60	
TraesCS3A02G189600	234210384			-0.05	
TraesCS3A02G189700	234215443			-0.37	
TraesCS3A02G189800	234940284			0.03	
TraesCS3A02G189900	235206120			-0.13	
TraesCS3A02G190000	235234560			-0.55	unknown
TraesCS3A02G190100	235370853			-0.19	
TraesCS3A02G190200	235381531			2.62	calmodulin-binding protein 25-like

TraesCS3A02G190300	235823604			-0.26	
TraesCS3A02G190400	237053956			0.00	
TraesCS3A02G190700	238676584			-0.08	
TraesCS3A02G190800	239957053			-0.20	
TraesCS3A02G191000	240355868			0.40	
TraesCS3A02G190900	240356072			-7.05	
TraesCS3A02G191100	241922344			0.40	
TraesCS3A02G191200	241924020			-0.26	
TraesCS3A02G191300	243862105			-0.54	
TraesCS3A02G191400	243870533			-0.23	
TraesCS3A02G191500	243873796			1.65	CO(2)-response secreted protease-like isoform X1
TraesCS3A02G191600	244350805			-6.76	
TraesCS3A02G191700	244482474			-0.66	
TraesCS3A02G191800	245598680			-0.28	
TraesCS3A02G191900	246041212			-0.31	
TraesCS3A02G192000	247729247			-0.03	
TraesCS3A02G192100	248218357			0.09	
TraesCS3A02G192200	248545758			0.12	
TraesCS3A02G192300	249746082			0.00	
TraesCS3A02G192400	249853150			-0.17	
TraesCS3A02G192500	251317981			-0.37	
TraesCS3A02G192600	256234246			0.01	
TraesCS3A02G192700	256865246			-0.19	
TraesCS3A02G192800	259063611			0.00	
TraesCS3A02G192900	259324185			0.07	
TraesCS3A02G193000	259838202			0.00	
TraesCS3A02G193100	260066304			-0.05	
TraesCS3A02G193200	261348337			-0.20	
TraesCS3A02G193400	262522275			-0.21	
TraesCS3A02G193500	264023366			-0.13	
TraesCS3A02G193600	265171852			-0.18	

TraesCS3A02G193700	265537026			-0.33	
TraesCS3A02G193800	266450899			-0.23	
TraesCS3A02G193900	266687510			-0.17	
	266691232		BS00110129		
TraesCS3A02G194000	267040216			-0.20	
TraesCS3A02G194100	269203873			-0.46	
TraesCS3A02G194200	269391598			-0.23	
TraesCS3A02G194300	269648176			0.03	
TraesCS3A02G194500	271616977			0.00	
TraesCS3A02G194600	272769515			-0.38	
TraesCS3A02G194700	273159694			-0.56	
TraesCS3A02G194800	275274775			-0.79	ubiquitin-like modifier-activating enzyme 5 isoform X3
TraesCS3A02G194900	276474877			0.09	
TraesCS3A02G195100	277487239			0.13	
TraesCS3A02G195200	278734929			-0.04	
TraesCS3A02G195300	279435212			-0.13	
TraesCS3A02G195400	281183990			-0.02	
TraesCS3A02G195500	283093819			0.07	
TraesCS3A02G195600	288535826			-0.18	
TraesCS3A02G195700	290414357			-0.36	
TraesCS3A02G195800	291784149			0.00	
TraesCS3A02G195900	291787652			-0.32	
TraesCS3A02G196000	293060645			0.00	
TraesCS3A02G196100	293585683			0.61	
TraesCS3A02G196200	295947787			0.70	
TraesCS3A02G196300	296923209			-0.27	
TraesCS3A02G196400	298146055			-0.23	
TraesCS3A02G196500	298370388			9.28	unknown
TraesCS3A02G196600	299554908			-0.08	
TraesCS3A02G196700	301120494			-0.07	
TraesCS3A02G196800	302928187			0.99	

TraesCS3A02G196900	303529702			-0.35	
TraesCS3A02G197000	303829226			0.12	
TraesCS3A02G197100	304889974			0.00	
TraesCS3A02G197200	306859162			-0.05	
TraesCS3A02G197300	307913850			0.03	
TraesCS3A02G197400	308674350			0.00	
TraesCS3A02G197500	308675476			-0.01	
TraesCS3A02G197600	308701135			-0.17	
TraesCS3A02G197700	309337811			-0.24	
TraesCS3A02G197800	310690670			-0.22	
	310746858		**barc19**		
	414814170		BS00022129		
	414814542		BS00010947		
	487458080		BS00003837		
	514312171		BS00011170		
	526865255		GA2ox3-1		
	562351788		BS00011612		
	625789607		**wmc264**		
	642367061		BS00071428		
	732472437		BS00029569		

Supplementary Table 1.

The full High Confidence gene content for the 265 Mb region covering the whole Ht and Hd QTL regions. This was obtained using BioMart at https://plants.ensembl.org/Triticum_aestivum/Info/Index The Gene ID and the Start position in the IWGSC RefSeq v1.0 are indicated.

Selected Mapping Markers used in the QTL mapping (KASP and SSR) and the QTL regions they define for the Ht and Hd QTLs are shown.

The expression level ratio between Avalon and Cadenza is indicated (log2FoldChange). Gene function is shown, if available.

Supplementary Table 2

A. Yield and yield component data from Church Farm 2015-16

Lines	mean GY	mean GPSQM	mean TGW	GY SEMs	GRPSQM SEMs
3A.72	9.382	18785	49.9	0.433	5421
3A.21	9.814	19634	49.9	0.093	5659
3A.24	10.203	20742	47.9	0.291	691
3A.26	9.197	18849	48.7	0.639	5457
3A.29	10.462	21127	49.5	0.231	634
3A.35	9.722	19598	49.6	1.013	5745
3A.56	10.290	21431	48.1	0.522	1388
3A.75	10.320	20755	49.7	0.438	1118
NIL-A	9.524	19192	49.6	0.391	5545
NIL-C	10.648	21264	50.1	0.533	1228
Avalon	9.302	19239	48.3	0.570	5568
Cadenza	9.358	18694	49.9	0.526	703

B. **Single marker regression analysis for yield components from Church Farm 2015-16**

	Ht					***Hd***
**bp**	61343069	61348484		81477543	106434896	168837814
**Marker**	BS00003801	BS00022516	BS00034344	BS00022624	BS00022148	BS00021976
**GRYLD.pValue**	0.882	0.714	0.714	0.303	0.478	0.256
**GRYLD.effect**	0.0137	-0.0365	-0.0365	0.107	0.0777	0.107
**GRYLD.mean**	9.91	9.97	9.97	9.74	9.81	9.79
**TGRWT.pValue**	0.805	0.935	0.935	0.813	0.613	0.386
**TGRWT.effectB**	0.0381	-0.0142	-0.0142	-0.0496	-0.105	-0.145
**TGRWT.mean**	49.3	49.4	49.4	49.5	49.6	49.6
**GRPSQM.pValue**	0.756	0.953	0.953	0.0689	0.177	0.0672
**GRPSQM.effectB**	73.2	-15.8	-15.8	492	394	409
**GRPSQM.mean**	19900	20100	20100	19000	19300	19400

Supplementary Table S2. GY = Grain Yield (tonnes per hectare), GPSQM = grains per square metre, TGW= Thousand grain weight. SEM= standard error of the mean. Lines 3A.XX are recombinants at the 3A locus. NIL-A and NIL-C are the Cadenza Near Isogenic Lines carrying an Avalon and Cadenza introgression respectively and from which the recombinants are derived. Avalon and Cadenza are the parental varieties. bp refers to the position in the IWGSC RefSeq v1.0. Details of BS markers can be found at https://www.cerealsdb.uk.net/cerealgenomics/CerealsDB/indexNEW.php. *Ht* and *Hd* show peak QTL marker positions for height and heading.
